# The Protein Arginine Methyltransferases 1 and 5 affect Myc properties in glioblastoma stem cells

**DOI:** 10.1038/s41598-019-52291-6

**Published:** 2019-11-04

**Authors:** Annarita Favia, Luisa Salvatori, Simona Nanni, Lisa K. Iwamoto-Stohl, Sergio Valente, Antonello Mai, Fiorella Scagnoli, Rosaria Anna Fontanella, Pierangela Totta, Sergio Nasi, Barbara Illi

**Affiliations:** 1 Institute of Molecular Biology and Pathology - National Research Council (IBPM-CNR), Rome, Italy; 2 Institute of Medical Pathology, Università Cattolica del Sacro Cuore, Rome, Italy; 3 Fondazione Policlinico Universitario A. Gemelli IRCCS, Rome, Italy; 4 University of Cambridge, Cambridge, UK; 5 Department of Chemistry and Technologies of Drugs, Sapienza University of Rome, Rome, Italy; 6 Pasteur Institute, Cenci-Bolognetti Foundation, Sapienza University of Rome, Rome, Italy; 7 Department of Biology and Biotechnology “Charles Darwin”, Sapienza University of Rome, Rome, Italy; 8 Futura Stem Cells s.r.l., Rome, Italy

**Keywords:** Cancer stem cells, Epigenetics, Methylation

## Abstract

Protein Arginine (R) methylation is the most common post-translational methylation in mammalian cells. Protein Arginine Methyltransferases (PRMT) 1 and 5 dimethylate their substrates on R residues, asymmetrically and symmetrically, respectively. They are ubiquitously expressed and play fundamental roles in tumour malignancies, including glioblastoma multiforme (GBM) which presents largely deregulated Myc activity. Previously, we demonstrated that PRMT5 associates with Myc in GBM cells, modulating, at least in part, its transcriptional properties. Here we show that Myc/PRMT5 protein complex includes PRMT1, in both HEK293T and glioblastoma stem cells (GSCs). We demonstrate that Myc is both asymmetrically and symmetrically dimethylated by PRMT1 and PRMT5, respectively, and that these modifications differentially regulate its stability. Moreover, we show that the ratio between symmetrically and asymmetrically dimethylated Myc changes in GSCs grown in stem versus differentiating conditions. Finally, both PRMT1 and PRMT5 activity modulate Myc binding at its specific target promoters. To our knowledge, this is the first work reporting R asymmetrical and symmetrical dimethylation as novel Myc post-translational modifications, with different functional properties. This opens a completely unexplored field of investigation in Myc biology and suggests symmetrically dimethylated Myc species as novel diagnostic and prognostic markers and druggable therapeutic targets for GBM.

## Introduction

The Myc (c-myc) oncogene is a transcription factor required for pivotal cell functions^1^. It regulates cell proliferation, metabolism, stemness maintenance^1^ and binds to 10% of genomic loci, usually amplifying transcription from already active genes^2,3^. Myc expression and function are deregulated in a wide variety of cancers, including glioblastoma multiforme (GBM)^4,5^. Despite more than a decade of advances in surgery and chemo-/radiotherapy, GBM treatment options are disconcertingly very poor and patients life expectancy is very limited (14.6 to 18 months from diagnosis)^6^. GBM poor prognosis and deadly outcome seem to rely on the presence of tumour initiating cells, which share many molecular and biological features with embryonic and adult stem cells and thus are called glioblastoma stem cells (GSCs). They are difficult to eradicate by surgery and are resistant to conventional radio- and chemotherapy, allowing tumour to recur overtime^7^. They also present the so-called “Myc signature”^8^, emphasizing the essential role Myc plays in maintaining GSC properties.

Myc is regulated by a series of post-translational modifications: phosphorylation^9^, acetylation^10^, sumoylation^11^, ubiquitination and consequent degradation by the proteasome machinery^12^. These modifications have a precise role in modulating Myc activity and have been suggested as potential therapeutic targets^9,10^.

Methylation of arginine (R) residues is the most common methylation event in mammalian cells, which modifies protein interacting properties, ruling a large amount of signaling pathways^13^. Three distinct types of methylated Rs occur in mammalians. Asymmetrically dimethylated arginine (ADMA) is the most frequent, followed by symmetrically dimethylated (SDMA) and monomethylated arginine (MMA). The enzymes responsible for R methylation are the Protein Arginine Methyltransferases (PRMTs). Six proteins have been reported to undoubtedly possess the R methyltransferase catalytic domain (PRMT1, 3, 4, 5, 6 and 8). PRMT2, 7 and 9 have sequence similarities with the other PRMTs and are supposed to possess an equivalent enzymatic activity^13^. Depending on the position of the methyl groups on R terminal guanidino nitrogens, PRMTs are, basically, classified into two types: type I PRMTs (PRMT1, 3, 4, 6 and 8) are responsible for MMA or ADMA whereas the type II PRMT5 catalyzes MMA and SDMA^13^. PRMT1 is the major methyltransferase in mammalian cells. It has a wide variety of substrate specificity, from histone to non-histone proteins^14^. From a transcriptional point of view, it is known as an activator, since the occurrence of asymmetrically (AS) dimethylated R3 on histone H4 residues (H4R3me2as) is associated with transcriptionally active chromatin^15^. PRMT1 is deregulated in a wide variety of cancer types, e.g. pancreatic adenocarcinoma^16^, gastric^17^ and lung cancer^18^. It controls the epithelial-mesenchymal transition (EMT) in cancer cells^19^, while playing a major role in muscle^20^ and B cell differentiation^21^. PRMT5 has received a greater attention and has been suggested as a putative druggable target in a wide variety of tumours, including GBM^22^. Like PRMT1, it has multiple nuclear and cytoplasmic targets and catalyzes the symmetrical (S) dimethylation of H4R3 and H3R8, usually repressing transcription^23^, and of H3R2, activating transcription^24^. PRMT5 is involved in a variety of cellular processes, such as neurogenesis^25^, myogenesis^26^, somatic cell reprogramming^27^ and GSCs self-renewal^28^. Interestingly, PRMT1 and PRMT5 have been shown to interact in GBM cells, being PRMT1 the major responsible for the activation of cancer-related genes^29^. They also catalyze the S- and AS-dimethylation of NMyc^30,31^ and, very recently, it has been shown that PRMT1 is required for p300 recruitment to Myc-regulated gene promoters^32^.

We previously demonstrated that PRMT5 interacts with Myc in glioblastoma cells, including GSCs, and that PRMT5 participates, at least in part, in the activation of Myc target genes^33^. Here we show that PRMT1 associates with Myc/PRMT5 in both HEK293T cells and GSCs and that Myc is both S- and AS-dimethylated by PRMT5 and PRMT1, respectively, both *in vitro* and in living cells. At the functional level, S-dimethylation protects Myc from degradation, while AS-dimethylation ensure Myc proper turnover. Finally, the inhibition of either PRMT1 or PRMT5 activity affects Myc recruitment at promoters and has a profound effect on GSCs biological functions, such as neurospheres formation and differentiation capacity. These findings represent the first demonstration in GSCs of the presence of differentially dimethylated Myc species, with distinct properties, opening a completely new field of investigation in Myc-dependent GBM biology. Further, they support the hypothesis that acting on S-Myc post-translational modification may represent a possible route to control its function.

## Results

### Myc interacts with PRMT1 and PRMT5

**Figure 1:**
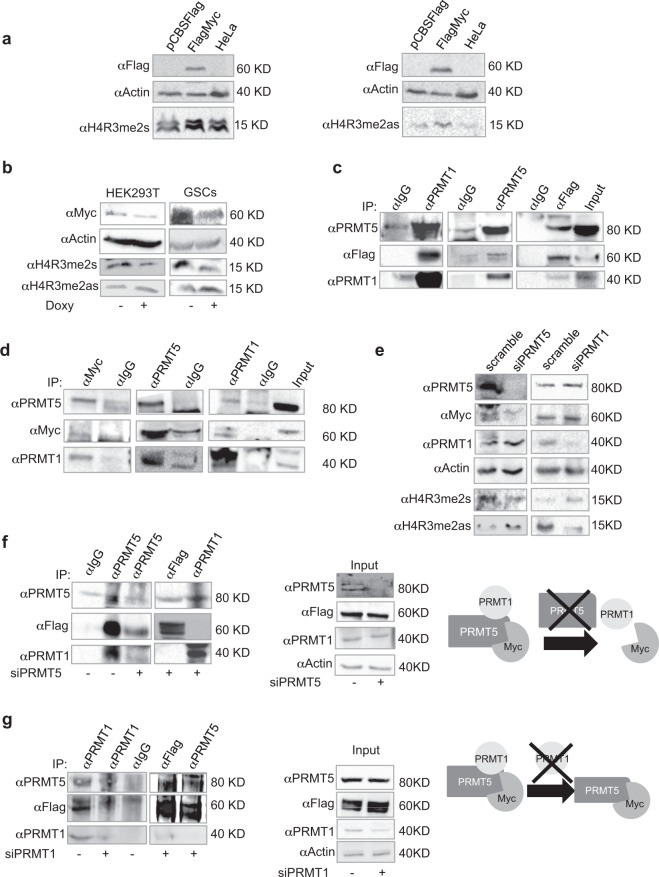
Myc/PRMT5/ PRMT1 complex. (**a**) Western blot. HEK293T cells were transfected with an empty or a FlagMyc expression vector. After 48 hrs, proteins were resolved onto a 12% polyacrylamide gel. β-actin was used as loading control. Uncropped images are shown in Supplementary Fig. S1a. (**b**) Western blot. Both HEK293T cells and GSCs were infected with a doxycycline inducible lentivirus carrying a shRNA against Myc (shMyc). After 48 hrs from doxycycline treatment, cells were lysed and proteins resolved onto a 12% polyacrilamide gel. Uncropped images are shown in Supplementary Fig. S1b. (**c,d**) Immunoprecipitations. FlagMyc/HEK293T cells and GSCs underwent reciprocal immunoprecipitation by using anti-Flag, anti-Myc, anti-PRMT1 and anti-PRMT5 antibodies (and control IgGs). Uncropped images are shown in Supplementary Fig. S1c,d. (**e**) Western blot. HEK293T cells were transfected with a scrambled siRNA or a pool of siRNAs against PRMT5 or PRMT1. Uncropped images are shown in Supplementary Fig. S1e. (**f**) Immunoprecipitation. HEK293T cells were transfected with a scrambled siRNA or a siRNAs pool against PRMT5. The day after, cells were transfected again with the FlagMyc expression vector. After further 48 hrs cells were immunoprecipitated with anti-PRMT5, anti-PRMT1 or anti-Flag antibodies (or control IgGs). Input is shown in the middle panel. The cartoon on the right panel outlines immunoprecipitation results. Uncropped images are shown in Supplementary Fig. S1f. (**g**) Immunoprecipitation experiments as in (**f**) in cells partially depleted of PRMT1 (see input, middle panel). The right panel outlines immunoprecipitation results. Uncropped images are shown in Supplementary Fig. S1g.

We have previously shown that Myc induces S-dimethylation of R3 on histone H4 (H4R3me2s, Fig. 1a, left and ref.^33^) and associates with PRMT5 in both HEK293T and glioblastoma cells^33^. Since PRMT5 and PRMT1 were found associated in GBM cells^29^, we sought to determine whether Myc was able to promote also AS-dimethylation of R3 on histone H4 (H4R3me2as). To this aim, HEK293T cells were transfected with either a Flag-tagged Myc construct (FlagMyc/HEK293T) or an empty vector and the level of H4R3me2as was detected by western blot. Figure 1a, right, shows H4R3me2as induction in the presence of FlagMyc construct. We reasoned that these histone modifications should decrease by Myc interference. However, in both HEK293T and mesenchymal GSCs^33,34^ transduced with a lentiviral, doxycycline inducible shRNA against Myc (shMyc), the level of H4R3me2s were reduced, while H4R3me2as increased (Fig. 1b), suggesting that impairing Myc-dependent PRMT5 activity is still sufficient to make H4R3 available for PRMT1 activity. Based on these data, we asked whether PRMT1, PRMT5 and Myc may interact. A series of reciprocal immunoprecipitation experiments, performed in FlagMyc/HEK293T cells, showed that FlagMyc associates with both PRMT5 and PRMT1 (Fig. 1c). No interactions were observed by transfecting the CBS-Flag vector alone, as expected (not shown). Consistently, the same result was obtained, at the endogenous level in GSCs (Fig. 1d). Overall, these data validate PRMT5/Myc interaction^33^ and indicate PRMT1 as a novel partner in this protein complex.

### PRMT5 is required for the formation of Myc/PRMT5/PRMT1 protein complex

We next wondered which protein member was necessary for complex assembly. Therefore, PRMT5 and PRMT1 expression was blunted by specific siRNAs in HEK293T cells (Fig. 1e). In siPRMT5/HEK293T cells, PRMT5 depletion was associated with a decrease in H4R3me2s levels, as expected, and with an increase in H4R3me2as, underlying the competition between PRMT5 and PRMT1 for the same histone substrate. Intriguingly, Myc protein also decreased. No effect on PRMT1 expression was observed. In siPRMT1/HEK293T cells, PRMT1 decreased together with H4R3me2as levels, as expected, while H4R3me2s increased. Myc protein slightly increased, while no effect on PRMT5 expression was detected. Although PRMT5 depletion was incomplete, Fig. 1f shows that the anti-Flag antibody did not co-immunoprecipitate PRMT1; accordingly, the anti-PRMT1 antibody did not co-immunoprecipitate the FlagMyc protein, as outlined in the model shown in the right panel. In cells partially depleted of PRMT1 (Fig. 1g) we were still able to observe a residual amount of PRMT5/PRMT1/FlagMyc complex, when an anti-PRMT1 antibody was used for immunoprecipitation. However, in anti-Flag and anti-PRMT5 immunoprecipitates, PRMT1 was undetectable, whereas PRMT5 and FlagMyc still interacted (see the scheme in the right panel). Overall, these data suggest that Myc/PRMT1 binding is mediated by PRMT5 in FlagMyc/HEK293T cells.

### Myc is both symmetrically and asymmetrically dimethylated

**Figure 2:**
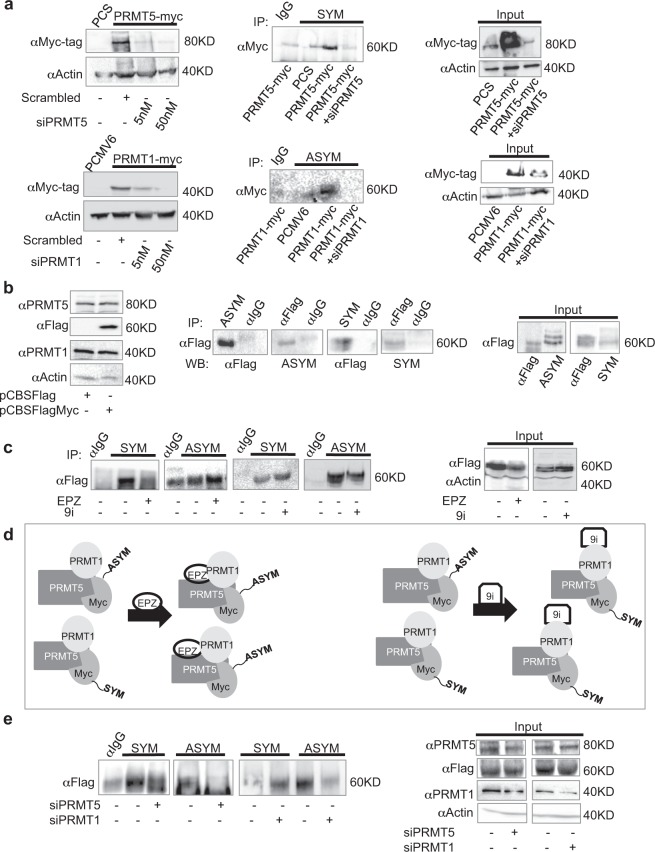
Symmetrical and asymmetrical Myc dimethylation. (**a**) Top, left. Western blot showing the efficacy of a siPRMT5 in inhibiting the expression of PRMT5-6myc (PRMT5-myc in the figure). Top, right. *In vitro* methylation assay showing S-dimethylation of a recombinant Myc protein in the presence of an overexpressed PRMT5. Bottom, left. Western blot showing the efficacy of a siPRMT1 in inhibiting the expression of PRMT1-myc-Dkk (PRMT1-myc in the figure). Bottom, right. *In vitro* methylation assay showing AS-dimethylation of a recombinant Myc protein in the presence of an overexpressed PRMT1. Uncropped images are shown in Supplementary Fig. S2a. (**b**) Left. Western blot analysis showing PRMT1 and PRMT5 expression levels upon FlagMyc transfection in HEK293T cells. Middle. FlagMyc/HEK293T cells underwent immunoprecipitation with control IgG or ASYM24, SYM10 and anti-Flag antibodies. Western blot were performed by using anti-Flag antibody for ASYM24 and SYM10 immunoprecipitations and ASYM24 or SYM10 antibodies for anti-Flag immunoprecipitation. Right. Western blot showing the input of the immunoprecipitations. Uncropped images are shown in Supplementary Fig. S2b. (**c**) Left. FlagMyc/HEK293T cells were treated for 24 hrs with EPZ015666 or 9i. After additional 24 hrs, immunoprecipitations were performed by using either SYM10 or ASYM24 antibody. Uncropped images are shown in Supplementary Fig. S2c. (**d**) The model depicts the competition between PRMT1 and PRMT5 for Myc. Inhibiting PRMT5 activity with EPZ015666 restrained FlagMyc S-dimethylation, while enhancing AS-dimethylation (left). The opposite was obtained by inhibiting PRMT1 (right). (**e**) HEK293T cells were transfected with a pool of specific siRNAs against either PRMT5 or PRMT1. The day after, cells were transfected with pCBS-FlagMyc. 48 hrs later, cells underwent immunoprecipitation with SYM10 or ASYM24 antibodies. Uncropped images are shown in Supplementary Fig. S2d. Abbreviations: SYM = SYM10; ASYM = ASYM24.

We wondered whether Myc was dimethylated by both PRMT5 and PRMT1. To ask this question, *in vitro* methylation assays were performed in HEK293T cells, overexpressing either myc-tagged PRMT5 or PRMT1, in the presence or absence of specific siRNAs (Fig. 2a). S- or AS-dimethylation of a recombinant human Myc protein was detected by immunoprecipitation experiments using antibodies recognizing either S- (SYM10 antibody) or AS- (ASYM24 antibody) dimethylated R residues. The recombinant human Myc was S-dimethylated in the presence of overexpressed PRMT5. S-dimethylation was restrained in the presence of a pool of PRMT5-specific siRNAs, as expected (Fig. 2a, top). Recombinant Myc AS-dimethylation was detected in the presence of a PRMT1-myc-Dkk overexpressed plasmid. No signal was observed in the presence of a specific siPRMT1 pool (Fig. 2a, bottom).

Moreover, protein extracts from HEK293T/FlagMyc cells were reciprocally immunoprecipitated by using SYM10 or ASYM24 or anti Flag antibodies. Western blots were performed with anti-Flag or SYM10 and ASYM24 antibodies. Figure 2b, middle panel, shows the presence of both S and AS- dimethylated FlagMyc in HEK293T cells. HEK293T cells transfected with pCBSFlag vector alone represented the negative control (not shown). To confirm that S- and AS-Myc dimethylation were PRMT5 and PRMT1-dependent, respectively, HEK293T/FlagMyc cells were treated for 24 hours with either 5 μM EPZ015666 – a commercially available PRMT5 specific inhibitor – or 3 μM 1,5‐bis(3,5‐Br_2_‐4‐hydroxyphenyl)‐1,4‐pentadien‐3‐one, hereafter named inhibitor 9 (9i), which specifically inhibits PRMT1 at this concentration^35^. Thereafter, immunoprecipitation experiments were performed with SYM10 and ASYM24 antibodies. Figure 2c, left, shows that PRMT5 inhibition impairs FlagMyc S-dimethylation, as expected. Conversely, PRMT5 inhibition increases the level of AS-dimethylated FlagMyc. These results were mirrored by similar experiments performed on cells treated with 9i. Indeed, PRMT1 inhibition reduced the level of AS-FlagMyc, while enhancing S-FlagMyc (Fig. 2c, left and scheme in d). In siPRMT5 transfected HEK293T/FlagMyc cells, S-FlagMyc was found at low levels, as expected. Interestingly, also AS-FlagMyc decreased (Fig. 2e). This is not surprising, as FlagMyc/PRMT1 interaction requires the presence of PRMT5 in HEK293T cells (Fig. 1f). In siPRMT1 transfected HEK293T/FlagMyc cells (Fig. 2e) we detected higher levels of S-FlagMyc, whereas AS-FlagMyc was severely impaired, confirming the binding of PRMT5 and Myc in the absence of PRMT1, as previously shown in Fig. 1g.

### PRMT1 and PRMT5 modulate Myc stability

**Figure 3:**
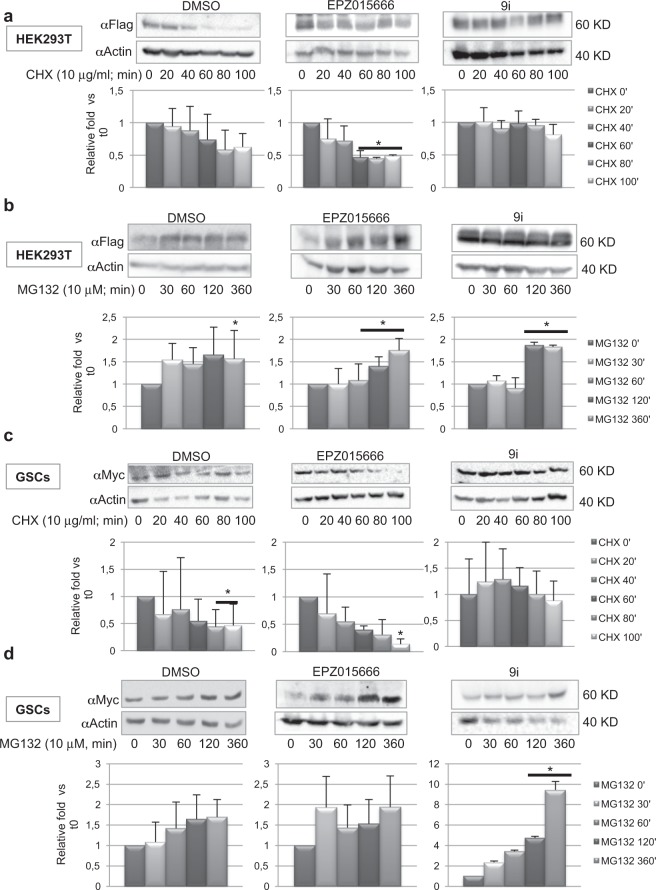
PRMT1 and PRMT5 control Myc protein stability. FlagMyc/HEK293T cells (**a**) and GSCs (**c)** were treated with either DMSO, EPZ015666 or 9i for 24 hrs. Thereafter, cycloheximide was added along a time course between 0 and 100 minutes and western blot analysis was performed. FlagMyc/HEK293T cells (**b**) and GSCs (**d**) were treated with either EPZ01566 or 9i for 24 hrs. Thereafter, MG132 was added along a time course between 0 and 360 minutes and western blot analysis was performed. *p < 0.05 vs DMSO. Uncropped images are shown in Supplementary Fig. S3a–d.

It has been shown that both PRMT1 and PRMT5 stabilize NMyc protein^30,31^. We observed that upon PRMT5 interference Myc expression decreased, while in PRMT1-depleted cells, Myc levels slightly increased (Fig. 1e). The same results were obtained inhibiting PRMT5 or PRMT1 activity, respectively (Fig. 2c, right). Therefore, two sets of experiments were performed, in order to investigate whether Myc stability was affected by either PRMT5 or PRMT1 catalytic inhibition in HEK293T/FlagMyc cells and in GSCs. To this aim, cells were treated with 5 μM EPZ015666 and 3 μM 9i for 24 hrs. Thereafter, cycloheximide (CHX, Fig. 3a,c) or the proteasome inhibitor MG132 (Fig. 3b,d) were added to the culture medium, along a time course between 0 and 100 minutes (CHX) or 0 and 360 minutes (MG132). In HEK293T/FlagMyc cells, we found that PRMT5 inhibition produced a decrease in FlagMyc protein levels upon CHX administration (Fig. 3a, middle), when compared to the control vehicle. This effect was also confirmed, and more evident, on endogenous Myc in GSCs (Fig. 3c, middle). Importantly, the enhanced degradation of Myc protein in the presence of EPZ015666 was not responsible for the decrease in Myc S-dimethylation (Fig. 2c). Indeed, immunoprecipitation experiments revealed that MG132 treatment did not recover S-Myc protein levels but only total FlagMyc (Supplementary Fig. S6), indicating that the decrease in Myc S-dimethylation was due to PRMT5 catalytic inhibition, although a combined effect with proteasome degradation may also be hypothesized. On the contrary, PRMT1 inhibition stabilized both FlagMyc and endogenous Myc upon CHX treatment (Fig. 3a,c, right). Proteasome inhibition by MG132 produced a similar, slight, but statistically significant, increase of FlagMyc protein levels in EPZ015666 and 9i HEK293T/FlagMyc treated cells (Fig. 3b). In GSCs, control vehicle and EPZ015666 produced similar effects upon MG132 exposure (Fig. 3d, left and middle, respectively). Conversely, 9i dramatically enhanced endogenous GSCs Myc protein content upon MG132 administration (Fig. 3d, right). These experiments suggest that in both HEK293T cells and GSCs, PRMT5 have a protective effect on Myc protein, while PRMT1 may allow its proper turnover or promote its degradation. However, these effects are particularly evident in GSCs.

### Myc is preferentially S-dimethylated in GSCs grown as neurospheres

**Figure 4:**
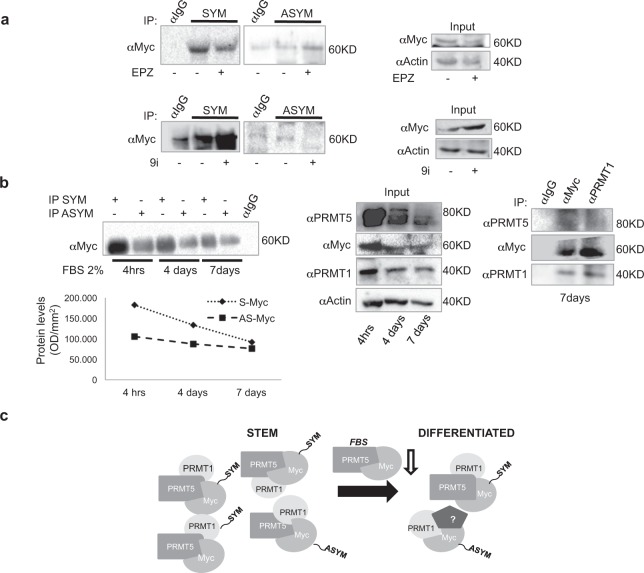
The ratio between Myc S-dimethylation and AS-dimethylation decreases upon cell differentiation. (**a**) GSCs were treated for 24 hrs with EPZ015666 (top) or 9i (bottom). Thereafter, immunoprecipitation experiments were performed by using either SYM10 or ASYM24 antibodies. Myc was revealed by an anti-Myc antibody. Inputs are shown on the right panels. Uncropped images are shown in Supplementary Fig. S4a. (**b**) Left. GSCs grown in 2% FBS containing medium along a time course between 4 hrs and 7 days. Thereafter, immunoprecipitation experiments were performed by using the SYM10 or the ASYM24 antibody, at each time point. Myc was revealed with an anti-Myc antibody. The densitometry, shown below the immunoprecipitation experiment, revealed a progressive reduction in S-Myc/AS-Myc ratio. Right panel. Immunoprecipitation experiment, showing the binding between Myc and PRMT1 at 7 days of FBS culture. PRMT5 was barely detectable. Input of both previous immunoprecipitation experiments is shown in the middle panel. Uncropped images are shown in Supplementary Fig. S4b. (**c**) The cartoon shows a model according to which S-Myc/AS-Myc ratio decreases upon GSCs differentiation, consistent with the reduced levels of PRMT5. Abbreviations: SYM = SYM10; ASYM = ASYM24; OD = optical density.

To evaluate endogenous Myc dimethylation in GSCs, neurospheres were treated for 24 hrs with either 5 μM EPZ015666 or 3 μM 9i. Cell extracts were immunoprecipitated with either SYM10 or ASYM24 antibody. An anti-Myc antibody was used to detect endogenous Myc. As shown in Fig. 4a, EPZ01566 treatment inhibited and 9i enhanced S-Myc, which represented the major Myc dimethylated species in GSCs. Consistently, 9i treatment decreased and EPZ015666 increased AS-Myc levels, which were, conversely, barely detectable in vehicle-treated GSCs. The presence of almost exclusively S-Myc species in GSCs grown as neurospheres prompted us to investigated whether Myc dimethylation pattern could change exposing GSCs to different stimuli. Indeed, we found that in GSCs grown in 2% FBS-containing medium, which promotes their astrocytic differentiation^36^, S-Myc levels were progressively reduced along a time course between 4 hours and 7 days, while AS-Myc remained quite stable (Fig. 4b, left). Consistently, almost total disappearance of PRMT5 and a scarce effect on PRMT1 expression upon FBS culture were observed, together with a reduction in Myc levels (Fig. 4b, middle). This also paralleled with immunoprecipitation experiments showing that Myc and PRMT1 could still interact in differentiating GSCs, while PRMT5 was only barely detected (Fig. 4b, right and model in panel c). In summary, these data suggest that also in GSCs Myc is both S- and AS-dimethylated and that PRMT5 and PRMT1 are competitors for Myc as a substrate. Furthermore, S-dimethylated Myc is the prevalent Myc species in GSCs grown as neurospheres whereas in differentiating GSCs its levels strongly decrease in a time-dependent manner. Differently, PRMT1 protein level and activity were weakly reduced during GSCs differentiation.

### PRMT1 and PRMT5 inhibition differentially modulates Myc recruitment at its target promoters in GSCs

**Figure 5:**
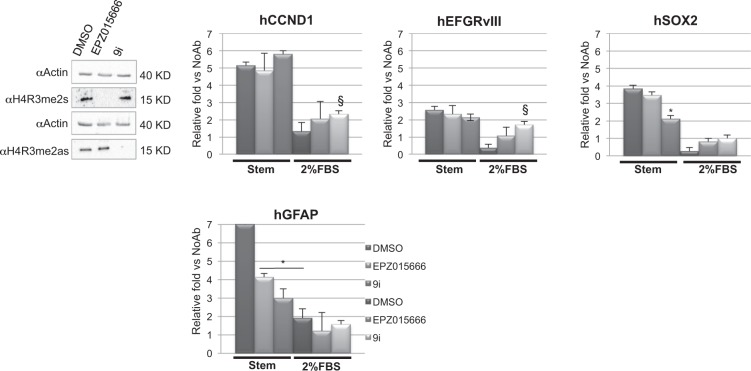
PRMT1 and PRMT5 affect Myc chromatin binding. ChIP experiments showing the effect of PRMT1 and PRMT5 catalytic inhibition on Myc binding at its target promoters. The left upper panel shows western blot experiments to validate EPZ015666 and 9i efficacy prior ChIPs. Uncropped images are shown in Supplementary Fig. S5a. *p < 0.05 vs DMSO, stem condition. ^§^p < 0.05 vs DMSO, 2% FBS.

To evaluate the effect of PRMT1 and PRMT5 on Myc binding at its specific chromatin loci in GSCs, ChIP experiments were performed, either in stem or differentiation conditions, in the presence or absence of EPZ015666 or 9i or control vehicle (Fig. 5). Two well known Myc promoter targets - cyclin D1 (CCND1) and the Epithelial Growth Factor Receptor variant III (EGFRvIII)^37^ - the stem gene promoters SRY-box 2 (SOX2), bound by Myc in GSCs^34^ together with the Glial Fibrillary Astrocytic Protein (GFAP) promoter^38^, were tested. In stem conditions, either PRMT5 or PRMT1 inhibition did not affect Myc binding on CCND1 and EGFRvIII promoters. Conversely, SOX2 promoter was sensitive only to PRMT1 inhibition, whereas, either PRMT5 or PRMT1 inhibition decreased Myc binding on GFAP promoter. Interestingly, Myc detached from all these promoters in differentiating control cells. PRMT5 inhibition had no effect on Myc recruitment in these experimental conditions, whereas 9i treatment restored Myc binding above control levels only on CCND1 and EGRvIII promoters. Altogether, these data address a role to both PRMT5 and PRMT1 catalytic activity in regulating Myc loading at promoters.

### PRMT5 affects GSCs cell cycle progression and neurospheres formation

**Figure 6:**
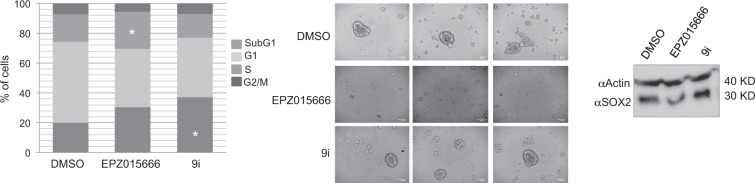
PRMT1 and PRMT5 inhibition differently affect GSCs cell cycle and self-renewal ability. Left. FACS analysis. GSCs were treated for 7 days with 5 μM EPZ015666, 3 μM 9i or control vehicle and then processed. Middle. Neurospheres formation. GSCs were cultured in the presence of EPZ015666, 9i or control vehicle for 10 days and monitored daily. Drugs were refreshed every 48 hrs. Right. Western blot showing the decrease of SOX2 protein level in EPZ015666-treated cells. Uncropped image is shown in Supplementary Fig. S5b. *p < 0.05 vs DMSO.

To evaluate in depth the effect of either PRMT1 or PRMT5 on GSCs biological properties, cell cycle, neurospheres formation, viability and apoptosis were evaluated. Cell cycle analysis (Fig. 6a, left) shows that a low, but statistically significant, number of GSCs treated for 7 days with EPZ015666 were arrested in S-phase, whereas an accumulation of cells in the subG1 phase was mostly observed upon 9i administration. Consistent with the role of PRMT5 in sustaining GSCs self-renewal^28^, neurospheres formation was extremely compromised after 10 days of EPZ015666 treatment, while 9i had no effect, when compared to control solvent (Fig. 6a, middle). This was paralleled by a decrease of SOX2 protein upon EPZ015666 exposure (Fig. 6a, right). Nevertheless, no differences in cell viability nor in apoptosis rate were observed in the presence of the two drugs, when compared to controls (not shown).

### PRMT1 affects GSCs differentiation

**Figure 7:**
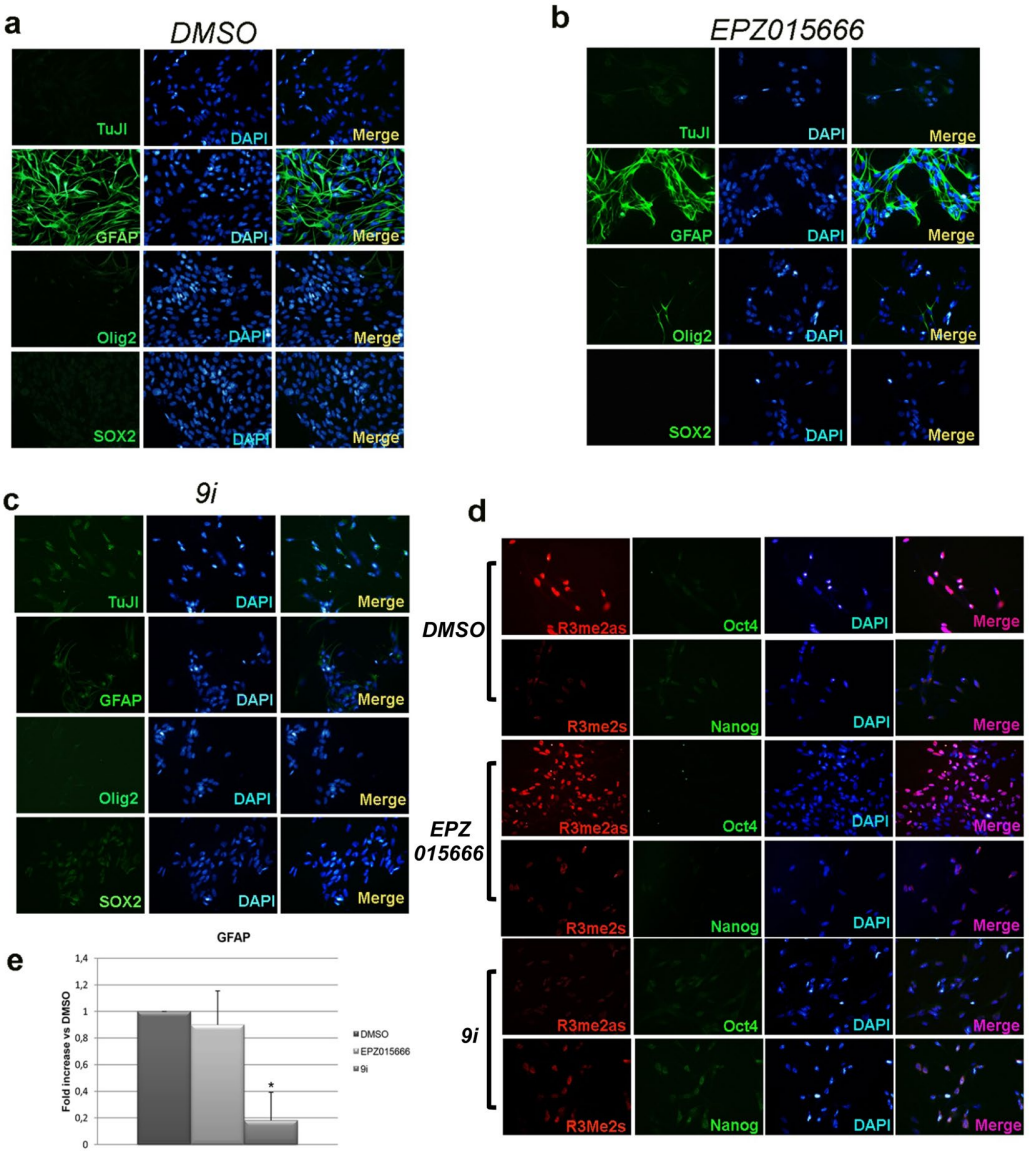
PRMT1 inhibition impairs GSCs astrocytic differentiation. (**a–d**) GSCs were pre-conditioned for 24 hrs in the presence of EPZ015666, 9i or control vehicle. The day after, cells were dissociated and plated in DMEM/F12 plus 2% FBS for 7 days. Drugs were refreshed every 48 hrs. Thereafter, cells were fixed and processed in immunofluorescence analyses. (**e**) Real time PCR for GFAP. *p < 0.05 vs DMSO.

As known, mesenchymal GSCs differentiate into astrocytes when cultured in the presence of foetal bovine serum (FBS)^36^ and we found that, in this condition, Myc is preferentially AS-dimethylated (Fig. 4b). Therefore we asked whether the inhibition of either PRMT1 or PRMT5 catalytic activity could modulate GSCs differentiation properties. To this aim, GSCs were pre-treated for 24 hrs with either EPZ015666 or 9i (or control vehicle); thereafter cells were shifted to FBS-containing medium and allowed to differentiate for 7 days. Drugs were refreshed every 48 hrs. Immunofluorescence analysis showed that control cells expressed GFAP but were negative for the stemness markers SOX2, Oct4, Nanog and Olig2^39^ (Fig. 7a). Also TuJI, a neuronal marker, was undetectable, as expected. Comparable data were obtained with EPZ015666 (Fig. 7b,d), whereas different results were observed upon PRMT1 inhibition. Differentiating GSCs exposed to 9i still did not express Olig2, TuJI, SOX2, Oct4 and Nanog proteins, and dramatically lost GFAP (Fig. 7c,d), as also confirmed by a decrease in its mRNA level, as shown in panel e. mRNAs encoding for the other protein markers analyzed were not modulated, upon neither PRMT5 nor PRMT1 inhibition (not shown). Furthermore, since PRMT1 activity is associated with astrocytic differentiation^40^, we monitored H4R3me2as and H4R3me2s levels. A prevalence of H4R3me2as was observed in control and EPZ015666 treated cells. Consistently, 9i blocked H4R3 AS-dimethylation, whereas a slight increase in H4R3me2s was observed (Fig. 7d). In summary, PRMT1 appears to support GSCs astrocytic differentiation, since its inhibition led to the loss of FBS-induced GFAP-positive cells. On the contrary, PRMT5 inhibition did not have any impact on GSCs commitment into the astrocytic lineage.

## Discussion

GBM still represents a great challenge for oncologists. Its high genetic, epigenetic and phenotypic heterogeneity have made impossible to identify valuable therapeutic routes, so far. PRMT1, PRMT5 and Myc have been all indicated as important players in GBM progression^5,41^. Specifically, Myc and PRMT5 have been proved as master regulators of GSC pluripotency^8,28^, while PRMT1 loss does not affect typical pluripotency markers^42^. Myc interacts with PRMT5^33^ and PRMT1 is required for p300 recruitment at Myc-regulated gene promoters^32^. Further, PRMT1 and PRMT5 interact in GBM^29^. However, no direct association between Myc, PRMT1 and PRMT5 has been reported. Here we show, for the first time, that Myc, PRMT5 and PRMT1 form a ternary complex, both in recipient HEK293T cells and GSCs (Fig. 1c,d). More importantly, we show that Myc is dimethylated by both PRMT1 and PRMT5 (Figs 2 and 4), being the presence of PRMT5 required for Myc/PRMT1 binding, at least in HEK293T cells (Figs 1f and 2e). Therefore, PRMT5 seems to function not only as a catalytic enzyme but also as a fundamental platform of the complex (Fig. 1f), at least in this cell system. Indeed, PRMT5 inhibition impaired also Myc AS-dimethylation (Fig. 2e). The inhibition of the catalytic activity of either PRMT1 or PRMT5 enhances Myc S- or AS-dimethylation, respectively (Figs 2b,c and 4a), without disrupting the complex and indicating that, as occurs for H4R3, they compete for Myc as a substrate. Myc dimethylation is consistent with the S- and AS-dimethylation of other transcription factors. Indeed, PRMT5 dimethylates and activates the Nuclear Factor kB (NF-kB) p65 subunit^43^, while AS-dimethylation by PRMT1 results in an impairment of the NF-kB RelA subunit binding to DNA^44^. Similarly, E2F-1 transcriptional specificity is regulated by either PRMT5-dependent S- or PRMT1-dependent AS-dimethylation, which are specifically induced by appropriate stimuli^45^. Further, NMyc, the neural-specific member of the Myc protein family, is both S- and AS-dimethylated by PRMT5^30^ and PRMT1^31^, respectively, and these modifications modulate protein stability. In this regard, our data show that, in both HEK293T cells and GSCs, PRMT5 stabilizes Myc (Fig. 3a,c, middle), while PRMT1 seems to regulate its proper turnover, as preventing PRMT1 catalytic activity maintained Myc protein levels constant upon *de novo* protein synthesis inhibition (Fig. 3a,c right) and induces a dramatic increase in GSCs Myc protein content, upon proteasome blockade (Fig. 3d, right). These results, together with the observation that GSCs possess an almost exclusively S-Myc when cultured in stem conditions (Fig. 4a), lead to the hypothesis that S-dimethylation may sustain Myc oncogenic function in GSCs. This is consistent with Myc and PRMT5 overexpression in GBM and with their role in supporting GSC stemness^4,28^. Inhibiting either PRMT1 or PRMT5 activity affects Myc recruitment at some of its target promoters (Fig. 5). In stem conditions, we observed that Myc recruitment was either unaffected (CCND1 and EGFRvIII promoters) or sensitive to PRMT1 inhibition (SOX2 promoter) or to both PRMT1 and PRMT5 inhibition (GFAP promoter), indicating that Myc binding activity is modulated by PRMT1 and PRMT5, either alone or in combination, in a gene-specific manner. Whether this phenomenon depends on Myc differential dimethylation, which may regulate its association to co-activators/co-repressors^32^, its promoter affinity and transcriptional specificity^45^ or on chromatin accessibility, which may impact Myc promoter binding efficacy, or on additive effects, is an issue still to be determined. Interestingly, upon differentiation Myc binding at promoters was strongly reduced and PRMT5 inhibition had a little impact on its recruitment, whereas PRMT1 inhibition rescued Myc binding at CCND1 and EGFRvIII promoters above control levels. Since S-Myc strongly decreases upon 7 days of differentiation (Fig. 4b), it may be supposed that AS-Myc may have a lower affinity for chromatin, as occurs for RelA^44^, and inhibiting AS-dimethylation may partially restore Myc binding capacity, at least on a subset of promoters.

At the functional level, inhibiting PRMT5 catalytic activity led to a statistically significant S-phase cell cycle accumulation of a small GSCs population and to the failure of neurospheres generation, as also reported by others^28^ (Fig. 6). On the other hand, PRMT1, which has no impact on GSCs maintenance, seems to dramatically affect GSCs differentiation (Fig. 7). Indeed, arginine methylation is critical for the onset of neural specific lineages during embryogenensis. In particular, PRMT1 activity is important for astrocytic differentiation of embryonic neural precursors^40^. Consistently, we have found that PRMT1 catalytic inhibition impairs GSCs differentiation into astrocytes (Fig. 7), while the loss of PRMT5 activity did not affect GSCs differentiation properties. Along the GSCs differentiation process, we observed a progressive decrease of S-Myc, which was paralleled by a decrease in PRMT5 and Myc protein levels, whereas AS-Myc remained stable (Fig. 4b). Therefore, upon differentiation, the S-Myc/AS-Myc ratio changes, in favour of AS-Myc. Consistently, we still detected Myc/PRMT1 binding after 7 days of differentiation (Fig. 4b, right), together with a residual amount of PRMT5 (Fig. 4b, middle). However, it has to be still determined whether Myc/PRMT1 interaction is direct or indirect and we cannot exclude the presence of other interactors to mediate Myc/PRMT1 association (see model in Fig. 4c).

We are aware that our observations lack mechanistic insights. In fact, the modifications in Myc binding ability at promoters (Fig. 5), GSCs cell cycle profile, failure of neurospheres formation (Fig. 6) and changes in the differentiation capability (Fig. 7) cannot be definitely addressed to S- or AS-Myc species. The identification of Myc dimethylated arginine(s), the production of specific, either unmethylatable or methyl-mimicking, Myc mutants, followed by depletion/reconstitution experiments to substitute the endogenous wild type Myc, is still missing and will be object of our future investigations.

In conclusion, our work reports, for the first time, that Myc associates with both PRMT1 and PRMT5 being differentially dimethylated. S-dimethylation protects Myc from degradation, while AS-dimethylation allows Myc proper turnover. We hypothesize S-Myc as typical of aggressive GSCs, as S-Myc/AS-Myc ratio decreases in differentiating, less aggressive, cells (Fig. 4b). In this view, S-Myc species may be suggested as a novel diagnostic and prognostic tool for GBM, once a specific antibody will be produced. Although thorough investigations are required to validate the hypothesis that S- or AS-Myc dimethylation may control Myc promoter specificity and/or binding (Fig. 5), this opens a completely new scenario on Myc epigenetic functions and may shed light onto some Myc properties which are not fully elucidated. Moreover, these findings suggest that acting on Myc S-dimethylation may represent a novel and unexplored therapeutic strategy for GBM, avoiding Myc complete silencing, which produces highly detrimental side effects in healthy cells.

## Methods

### Cell culture and treatments

HEK293T and mesenchymal Brain Tumour 168 Neural Stem Cells^33,36^ were cultured as described^33^. For differentiation experiments, GSCs were cultured in Dulbecco’s Modified Eagle Medium/F12 mixture (1:1), 1% Glutamine, 1% Penicillin/Streptomycin and 2% Foetal Bovine Serum (FBS, Merck, Darmstadt, Germany).

EPZ015666 (Merck) was administered at a concentration of 5 μM and the PRMT1 inhibitor 1,5‐bis(3,5‐Br_2_‐4‐hydroxyphenyl)‐1,4‐pentadien‐3‐one (9i) at 3 μM^35^. Control solvent was represented by dimethylsulfoxide (DMSO, Merck). For GSCs differentiation and neurospheres formation experiments, drugs were refreshed every 48 hrs. Cyclohexymide and MG132 (Merck) were 10 μg/ml and 10 μM, respectively.

### Western blot analyses

Cells were directly lysed in Laemmli buffer and western blots were performed as described^33^. Anti-H4R3me2s (cat. #ab5823; dilution 1:1000), anti-H4R3me2as (cat. #ab194683; dilution 1:1000), anti-PRMT5 (cat. #ab109451; dilution 1:1000) and anti-PRMT1 (cat. #ab3768; dilution 1:400) antibodies were from Abcam (Cambridge, UK). Anti-Myc (clone 9E10, cat. #sc-40; dilution 1:500) was from Santa Cruz Biotechnology (Dallas, TX, U.S.A). Anti-actinHRP (cat. #A3854; dilution 1:5000) was from Merck. Anti-SOX2 (cat. #MAB4343; dilution 1:500), SYM10 (cat. #07-412; dilution 1:1000) and ASYM24 (cat. #07-414; dilution 1:1000) antibodies were from Millipore (Burlington, MA, U.S.A.). Anti-Flag antibody (cat. #F1804; dilution 1:1000) was from Merck. Secondary anti-rabbit IgG (cat. #A0545; dilution 1:5000) and anti-mouse IgG (cat. #A9044; dilution 1:2000) were from Merck. In most cases, filters were directly cut after the blocking procedure, according to the molecular weight of the proteins of interest. Equipment and settings: images were captured with a ChemiDoc XRS+ Gel Imaging System (Bio-Rad, Hercules, CA, U.S.A) and the band intensity of western blot analysis was quantified using ImageJ software. When useful, contrast/brightness of western blot images were slightly adjusted with the Power Point software.

### Immunoprecipitation

For immunoprecipitation experiments, cells were lysed in 140 mM NaCl, 10 mM Tris pH 7.6, 1% Triton X-100, 0.1% sodium deoxycholate, 1 mM EDTA, protease and phosphatase inhibitors. Cells were incubated on ice for 30′, then centrifuged for 20′ at 4 °C, at 13000 rpm. Thereafter, supernatant (1–2 mg) underwent immunoprecipitation as described^33^, by using 4 μg of each antibody or control IgGs (mouse IgGs were from Santa Cruz, cat. #sc-2025; rabbit IgGs were from Merck, #cat. I8140). To avoid IgGs detection, either ImmunoCruz IP/WB Optima B, C, F, E (Santa Cruz Biotechnology; cat. #sc-45039, cat. #sc-45040, cat. #sc-45043, cat. #sc-45042, respectively) or the VeriBlot reagent (Abcam; #cat. ab131366 and #ab131368) were used in western blot, after the immunoprecipitation procedure, according to the manufacturer’s instructions.

### Plasmid transfection and lentiviral transduction

Plasmid transfections were performed by using Lipofectamine 2000 (ThermoFisher Scientific, Waltham, MA, U.S.A.) according to the manufacturer’s instructions. Plasmids were: pCBSFlagMyc^33^, pCMVPRMT5-6myc (courtesy of dr. Levi A.) and pCMV6PRMT1-myc-Dkk (Origene, Rockville, MD, U.S.A), together with the corresponding empty vectors. The shMyc insert was subcloned from the KRAB lentiviral vector into the Tet-on pSLIK lentivirus^33^. Lentiviral production and infection were performed as described^33^.

### siRNA transfection

siPRMT5, siPRMT1 (ON-Target Plus Human PRMT5 and PRMT1 siRNA, Dharmacon, Lafayette, CO, U.S.A.) and scrambled siRNAs (#sc-44230, Santa Cruz Biotechnology) were transfected by using the DHARMAFect reagent (Dharmacon), according to the manufacturer’s instructions.

### *In vitro* methylation assay

For *in vitro* methylation assays, HEK293T cells (3,5 × 10^6^) were transfected with 50 nM siRNAs (or scrambled siRNA) and the day after with either PRMT5-6myc or PRMT1-myc-Dkk plasmids or control vectors (10 μg). 48 hrs later, cells were lysed in 20 mM Hepes pH 7.9, 137 mM NaCl, 20% Glycerol, 1% Nonidet P40, 2 mM EDTA, protease and phosphatase inhibitors. Cell extracts were kept in rotation for 30′, at 4 °C and then centrifuged at 12000 rpm for 20′ at 4 °C. Cell extracts were pre-cleared with 5 μg of control IgGs, for 1 hr, on ice; thereafter, 20 μl of equilibrated protein A/G Plus agarose beads (Santa Cruz Biotechnology; cat. #sc-2003) were added and the mixture was kept in rotation for 30′, at +4 °C. and then centrifuged at 10000 rpm, at 4 °C. The supernatant was incubated with either anti-Myc antibody (Santa Cruz Biotechnology), or control IgG, for an overnight, at 4 °C (to purify either overexpressed PRMT5-6myc or PRMT1-myc-Dkk). The day after, 40 μl of equilibrated protein A/G Plus agarose beads were added and the mixture was incubated in rotation for 4 hrs, at 4 °C. Beads were extensively washed with lysis buffer by centrifuging at 3000 rpm, at 4 °C. Beads and bound PRMT5-6myc or PRMT1-myc-Dkk were resuspended in assay buffer (20 mM Tris HCl pH 7.5, 20% glycerol, 100 mM KCl, 1 mM DTT, 0.2 mM EDTA, protease and phosphatase inhibitors) and 500 ng of human recombinant c-Myc protein (Abcam; cat. #ab84132) plus 1 mM of S-adenosylmethionine (Merck) were added. The reaction mixture (100 μl) was incubated for 1 hr, at 35 °C. Thereafter, 400 μl of assay buffer were added, and dimethylated Myc was immunoprecipitated by incubating the reaction mixture with 4 μg of either SYM10 or ASYM24 antibody (Millipore), for an overnight, at 4 °C. The day after, 40 μl of equilibrated protein A/G Plus agarose beads were added and the mixture incubated for 4 hrs, in rotation, at 4 °C. After three washes in assay buffer, 20 μl of Laemmli buffer 2X were added and the immunocomplexes boiled for 5′. Dimethylated Myc was revealed by western blot.

### Immunofluorescence analyses

Immunofluorescence analyses were performed as described^33^. Anti-Olig2 (cat. #sc-293163; dilution 1:50), anti- TuJI (cat. #sc-51670; dilution 1:50), anti-Oct4 (cat. #sc-101534; dilution 1:50) antibodies were from Santa Cruz Biotechnology; anti-SOX2 (cat. #MAB4343; dilution 1:50) and anti-Nanog (clone 7F7.1, cat. #MABD24; dilution 1:50) were from Millipore. Anti-GFAP (cat. #ab7260; dilution 1:100), anti-H4R3me2s (cat. #ab5823; dilution 1:100) and anti-H4R3me2as (cat. #ab194683; dilution 1:100) were from Abcam. Secondary antibodies were donkey anti-rabbit IgG Alexa Fluor 555 (cat. #A-31572; dilution 1:100) and goat anti-mouse Alexa Fluor 488 (cat.# A-11029; dilution 1:100), both from Thermo Fisher Scientific.

### Cell cycle analysis

Cells were dissociated, counted, resuspended in PBS and then fixed in 70% ethanol for a minimum of 24 hrs. After three washes in PBS, cells were incubated with 50 μg/ml Propidium Iodide (Merck) plus 0.1 U/L RNAse (Merck) for 3 hrs at room temperature before FACS analysis by Coulter Epics XL flow cytometer (Beckman Coulter, Brea, CA, U.S.A.). Cells were gated using both area *vs* peak to eliminate doublets and forward *vs* side scatter to exclude debris. The percentage of cells in the different cycle phases and in subG1 (composed of dead cells and debris) was calculated using FACS Express5 Flow Research Edition software.

### Neurospheres formation

3 × 10^5^ GSCs were plated in duplicate into a six-well multi-plate. Cells were allowed to grow for 10 days, in the presence or absence of EPZ015666 or 9i or control vehicle. Neurospheres formation was monitored daily by a phase contrast microscope. Images were captured with the ZOE Fluorescent Cell Imager (Bio-Rad).

### Real time PCR

Real time PCR were performed as described^33^.

### Chromatin Immunoprecipitation

Chromatin immunoprecipitations were performed as described^46^.

### Oligonucleotides

ChIPs oligonucleotides were:

hEGFRv3prom_F GGCTGTTTGTGTCAAGCCTTTA

hEGFRv3prom_R TTGAAGCCAATGTGTGAAGCA

hSOX2prom_F AACGGACGTGCTGCCATT

hSOX2prom_R TCCCATTGTCCCGACGTAAA

hGFAPprom_F TGGGAATGAGGCCTAGTAGGAA

hGFAPprom_R GGGAATTTGGCGAAGAATGA

hCCND1prom_F GCTTTCCATTCAGAGGTGTGTTT

hCCND1prom_R CTACCTTGACCAGTCGGTCCTT

GFAP oligonucleotides used in real time PCR experiments were:

GFAP_F ACCAGGACCTGCTCAATGTC

GFAP_R ATCTCCACGGTCTTCACCAC

### Quantification and statistical analyses

All data are expressed as mean ± SEM, and significance (p value) was calculated using two-tailed paired Student’s t test; p values < 0.05 were considered statistically significant.

## Supplementary information

Supplementary info
