# Supplementary material for: The Protein Arginine Methyltransferases 1 and 5 affect Myc properties in glioblastoma stem cells
Source: Sci Rep. 2019 Nov 4;9:15925. doi: 10.1038/s41598-019-52291-6 (PMC6828805; doi:10.1038/s41598-019-52291-6)
Supplement: Supplementary file 1 — Supplementary info [file 41598_2019_52291_MOESM1_ESM.pdf]

MS SREP-19-11671B "The Protein Arginine Methylatransferases 1 and 5 affect Myc properties in glioblastoma stem cells"

Supplementary Figures

Annarita Favia, Luisa Salvatori, Simona Nanni, Lisa K. Iwamoto-Stohl, Sergio Valente, Antonello Mai, Fiorella Scagnoli, Rosaria Anna Fontanella, Pierangela Totta, Sergio Nasi, Barbara Illi.

Figure S1

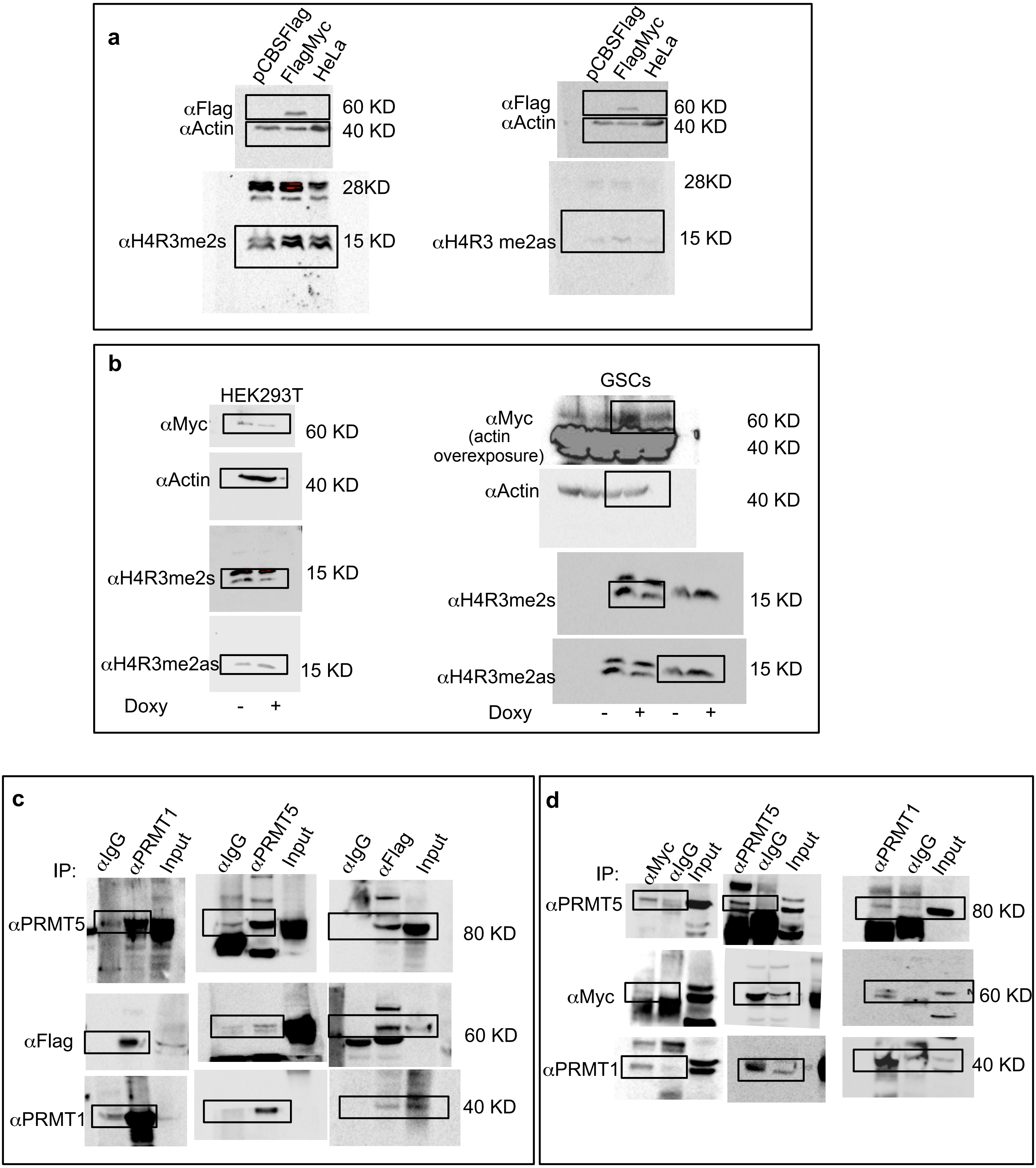
a
pCBSFlag
FlagMyc
HeLa
αFlag
αActin
60 KD
40 KD
28KD
αH4R3me2s
15 KD
αH4R3 me2as
b
HEK293T
αMyc
αActin
αH4R3me2s
αH4R3me2as
Doxy
- +
GSCs
αMyc
(actin
overexposure)
- + - +
c
IP:
αIgG
αPRMT1
Input
αPRMT5
αFlag
80 KD
d
αMyc
αIgG

Figure S1

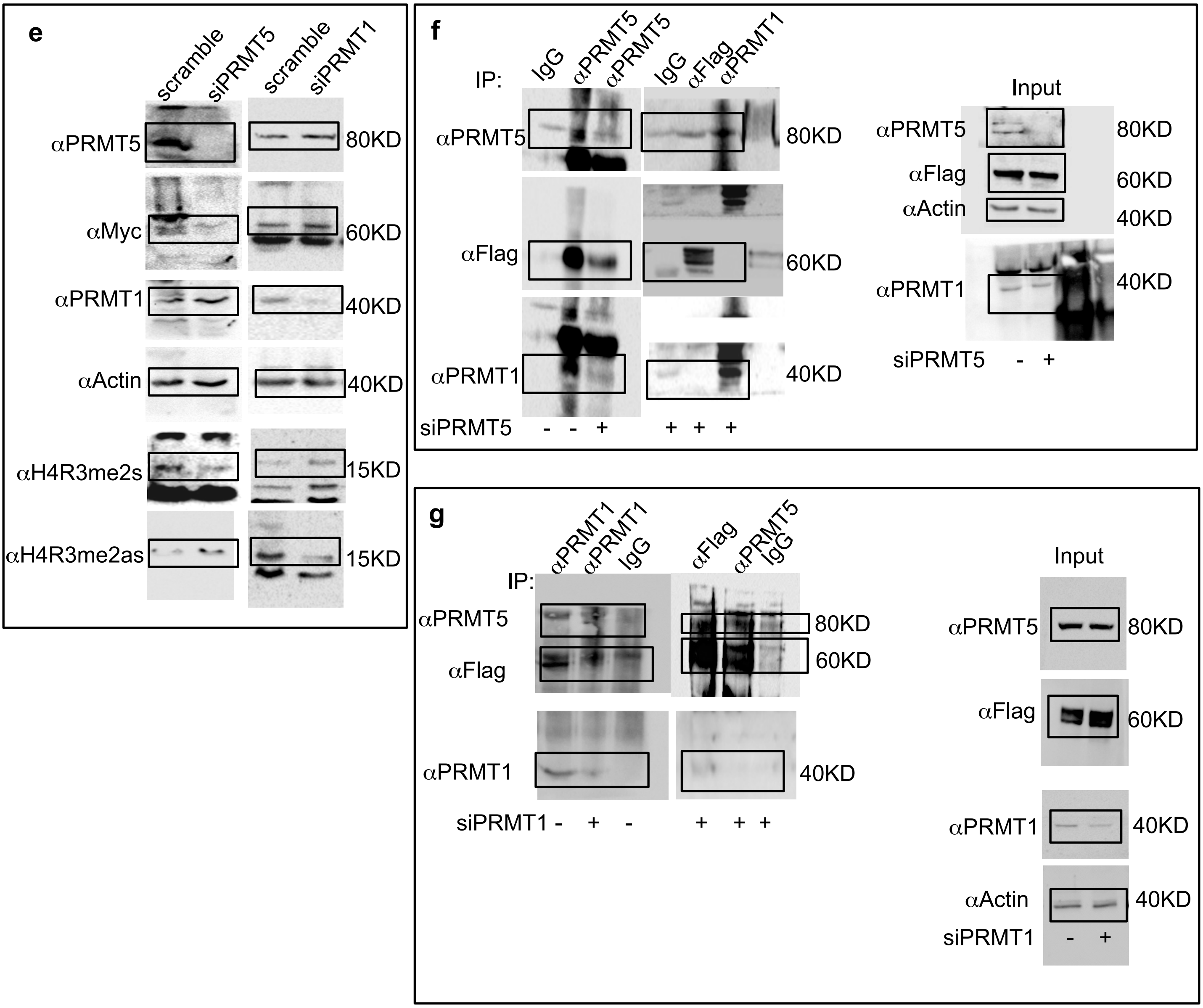

e
scramble
siPRMT5
scramble
siPRMT1
αPRMT5
80KD
αMyc
60KD
αPRMT1
40KD
αActin
40KD
αH4R3me2s
15KD
αH4R3me2as
15KD
f
IP:
IgG
αPRMT5
αPRMT5
IgG
αFlag
αPRMT1
αPRMT5
80KD
αFlag
60KD
αPRMT1
40KD
siPRMT5 - - + + + +
Input
αPRMT5
80KD
αFlag
60KD
αActin
40KD
αPRMT1
40KD
siPRMT5 - +
g
IP:
αPRMT1
αPRMT1
IgG
αFlag
αPRMT5
IgG
αPRMT5
80KD
αFlag
60KD
αPRMT1
40KD
siPRMT1 - + - + + +
Input
αPRMT5
80KD
αFlag
60KD
αPRMT1
40KD
αActin
40KD
siPRMT1 - +

Figure S2

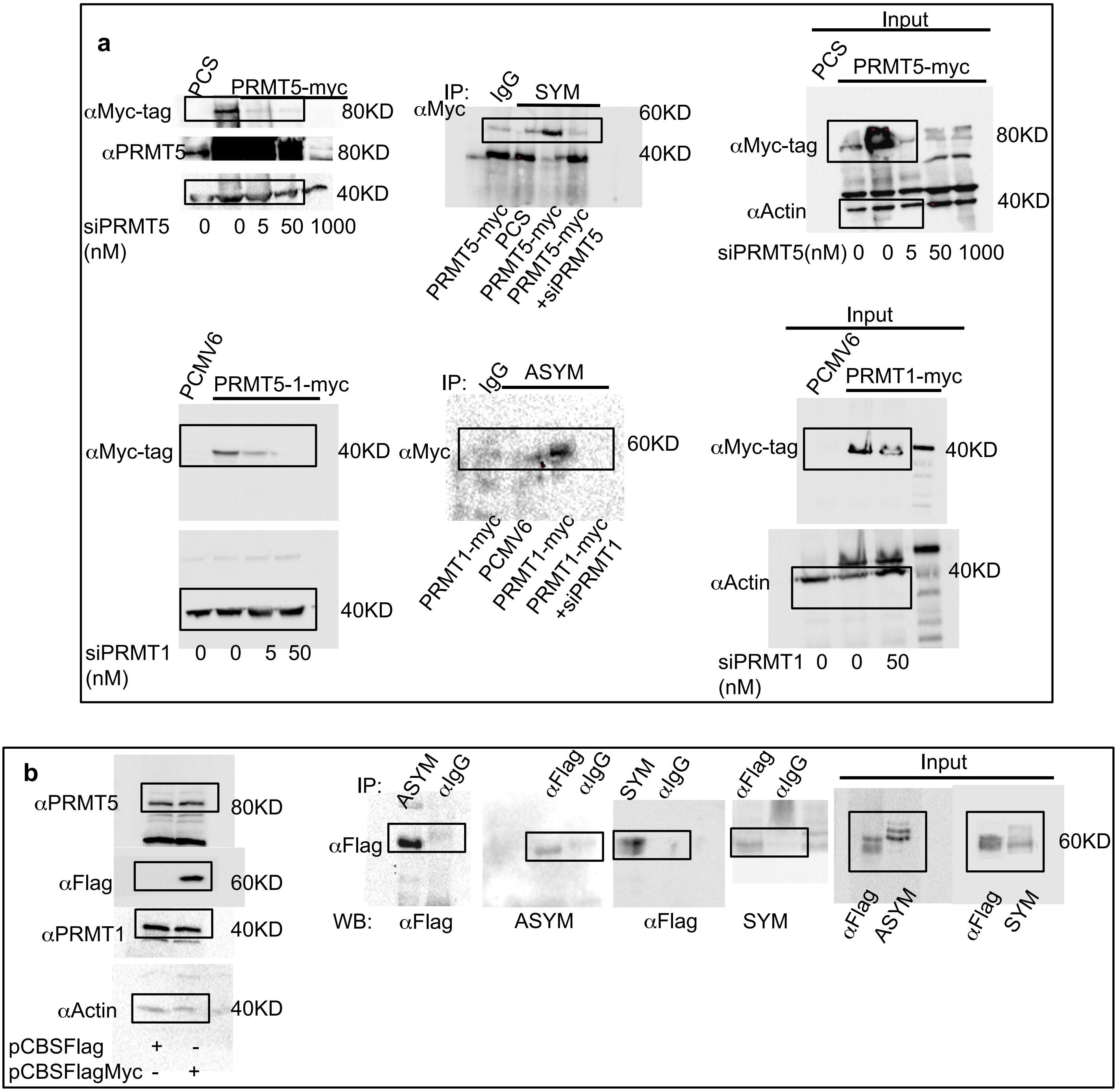
a
PCS
PRMT5-myc
αMyc-tag
80KD
αPRMT5
80KD
40KD
siPRMT5
(nM)
0 0 5 50 1000
IP: IgG SYM
αMyc
60KD
40KD
PRMT5-myc
PCS
PRMT5-myc
PRMT5-myc
+siPRMT5
Input
PCS
PRMT5-myc
αMyc-tag
80KD
αActin
40KD
siPRMT5(nM) 0 0 5 50 1000
PCMV6
PRMT5-1-myc
αMyc-tag
40KD
40KD
siPRMT1
(nM)
0 0 5 50
IP: IgG ASYM
αMyc
60KD
PRMT1-myc
PCMV6
PRMT1-myc
PRMT1-myc
+siPRMT1
Input
PCMV6
PRMT1-myc
αMyc-tag
40KD
αActin
40KD
siPRMT1
(nM)
0 0 50
b
αPRMT5
80KD
αFlag
60KD
αPRMT1
40KD
αActin
40KD
pCBSFlag + -
pCBSFlagMyc - +
IP: ASYM αIgG
αFlag
αFlag αIgG
SYM αIgG
αFlag αIgG
WB: αFlag
ASYM
αFlag
SYM
Input
αFlag
ASYM
αFlag
SYM
60KD

Figure S2

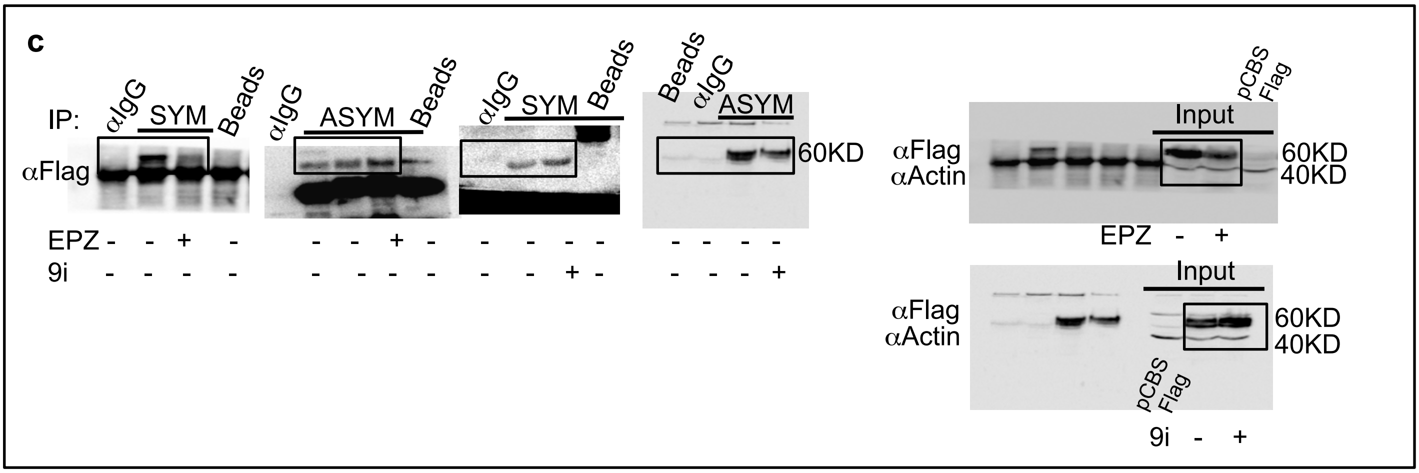
c
IP:
αIgG
SYM
Beads
αFlag
αIgG
ASYM
Beads
αIgG
SYM
Beads
Beads
αIgG
ASYM
60KD
EPZ
9i
αFlag
αActin
Input
pCBS Flag
60KD
40KD
EPZ - +
αFlag
αActin
Input
60KD
40KD
pCBS Flag
9i - +

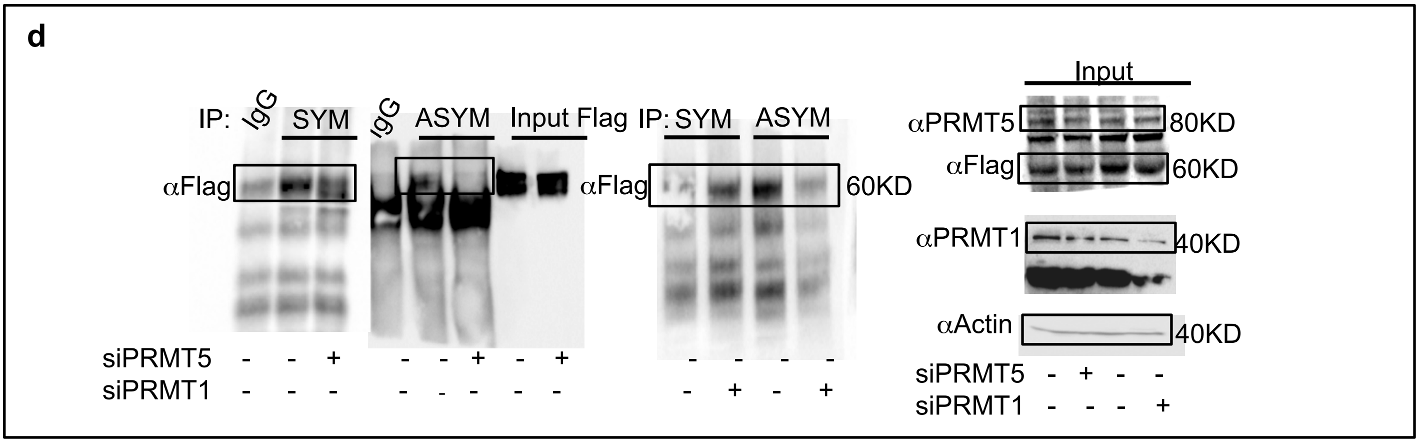
d
IP:
IgG
SYM
IgG
ASYM
Input Flag
IP: SYM
ASYM
αFlag
αFlag
60KD
siPRMT5
siPRMT1
Input
αPRMT5
80KD
αFlag
60KD
αPRMT1
40KD
αActin
40KD
siPRMT5
siPRMT1

Figure S3

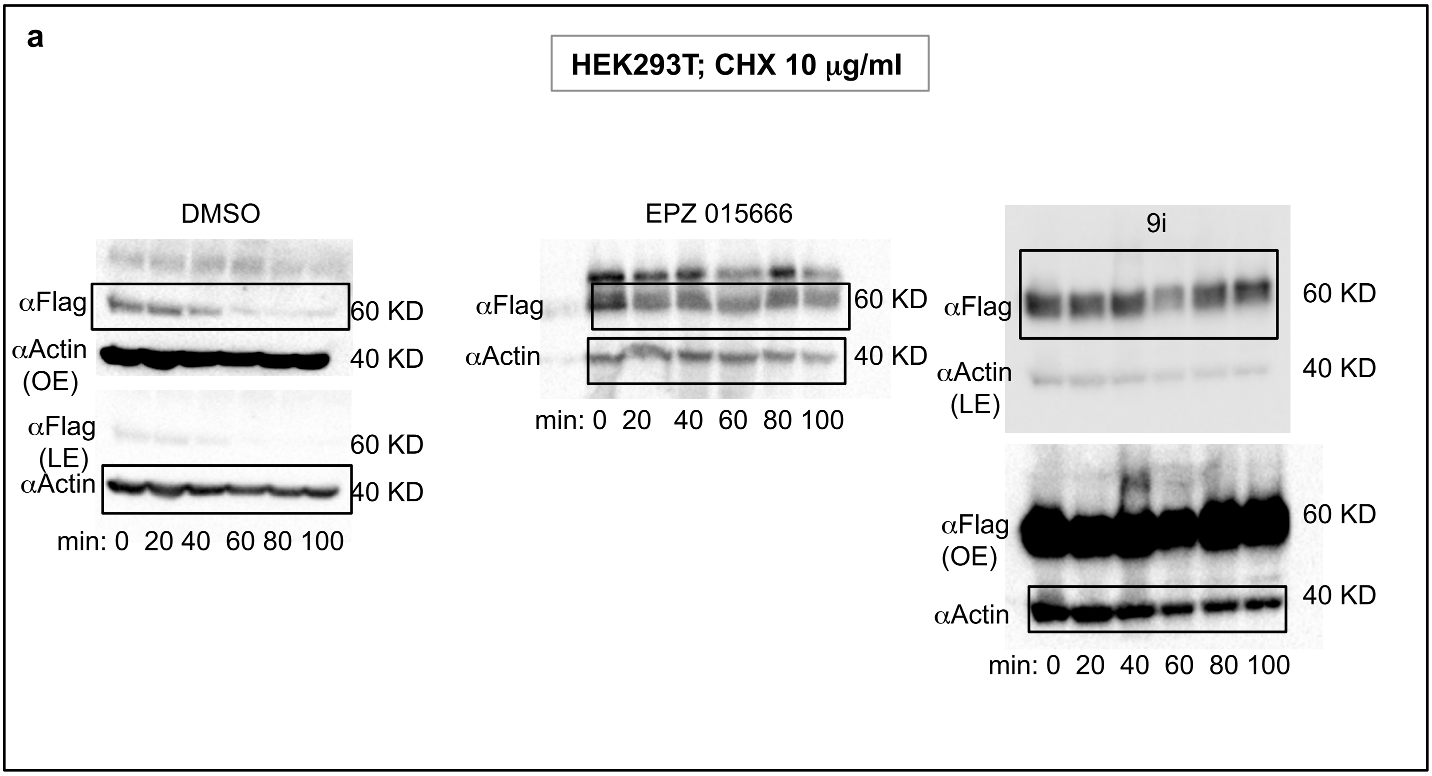

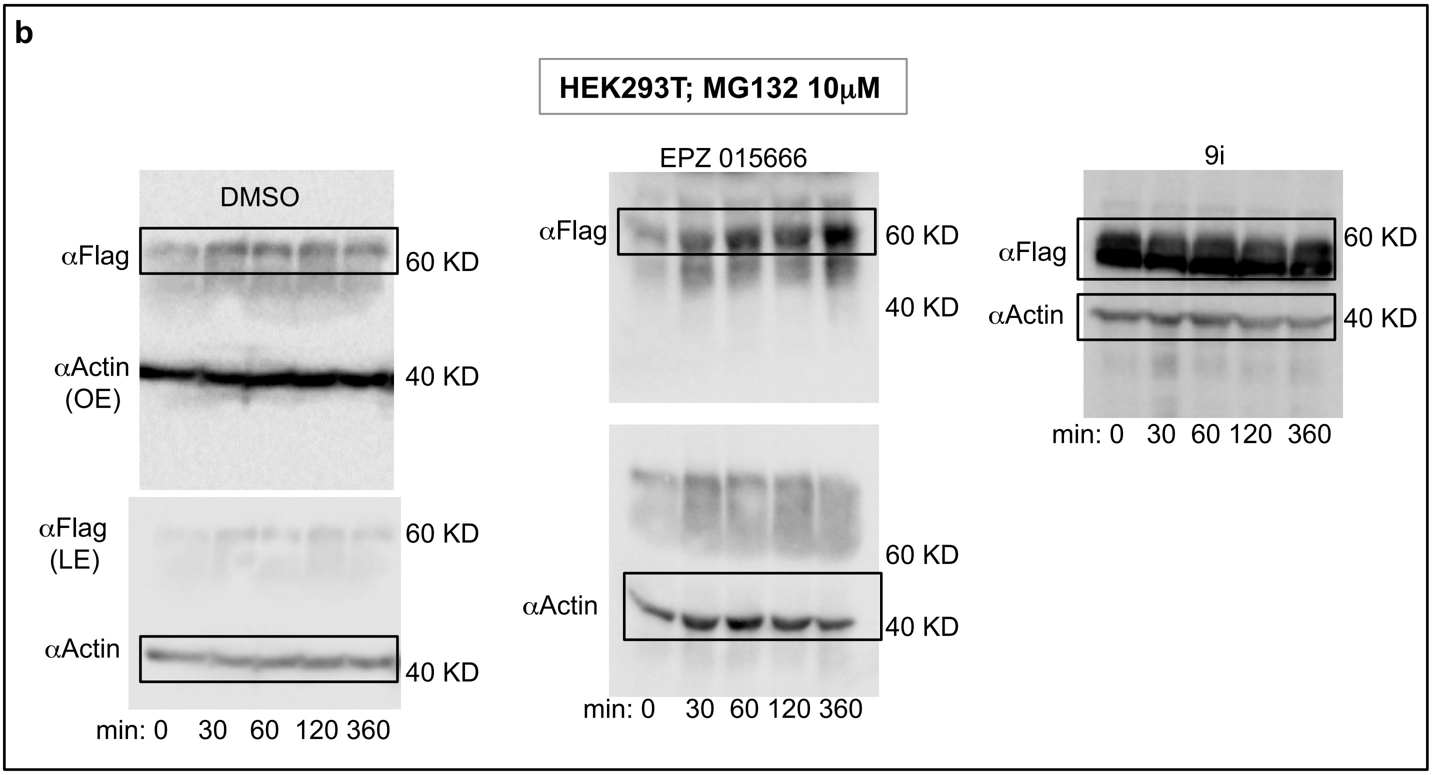

Abbreviations: OE = over exposure; LE = low exposure

Figure S3

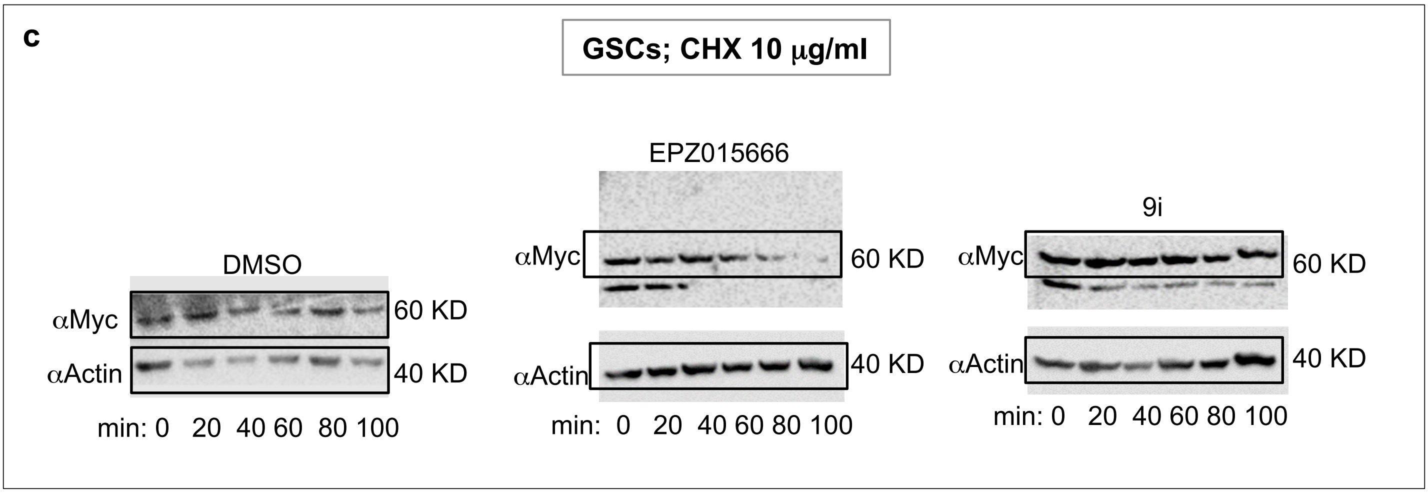
c
GSCs; CHX 10 μg/ml
DMSO
αMyc
αActin
60 KD
40 KD
min: 0 20 40 60 80 100
EPZ015666
αMyc
αActin
60 KD
40 KD
min: 0 20 40 60 80 100
9i
αMyc
αActin
60 KD
40 KD
min: 0 20 40 60 80 100

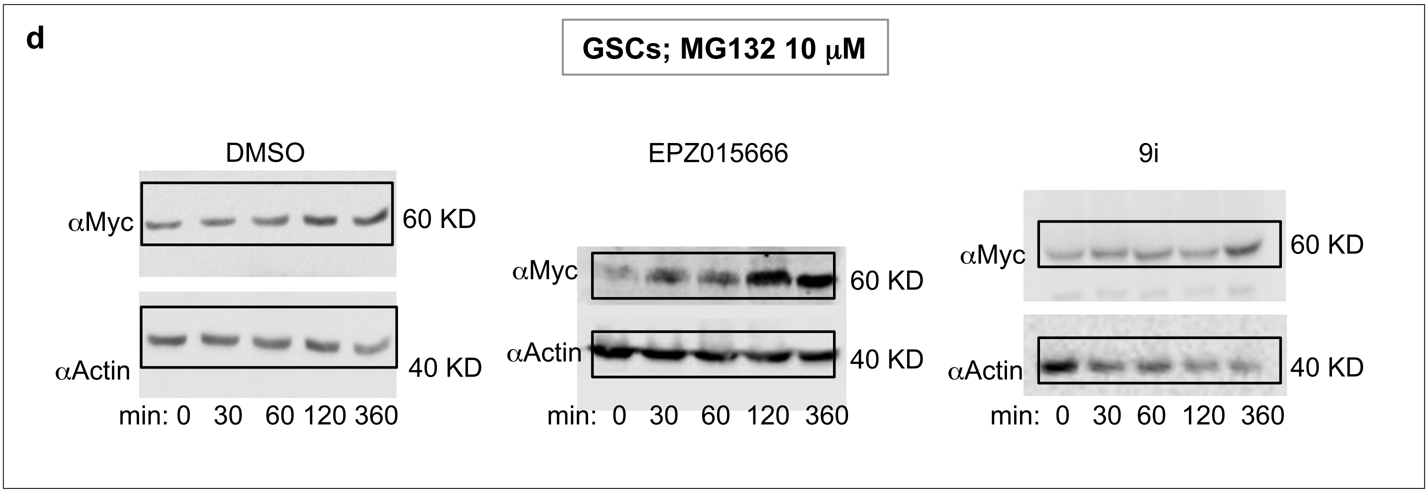
d
GSCs; MG132 10 μM
DMSO
αMyc
αActin
60 KD
40 KD
min: 0 30 60 120 360
EPZ015666
αMyc
αActin
60 KD
40 KD
min: 0 30 60 120 360
9i
αMyc
αActin
60 KD
40 KD
min: 0 30 60 120 360

Figure S4

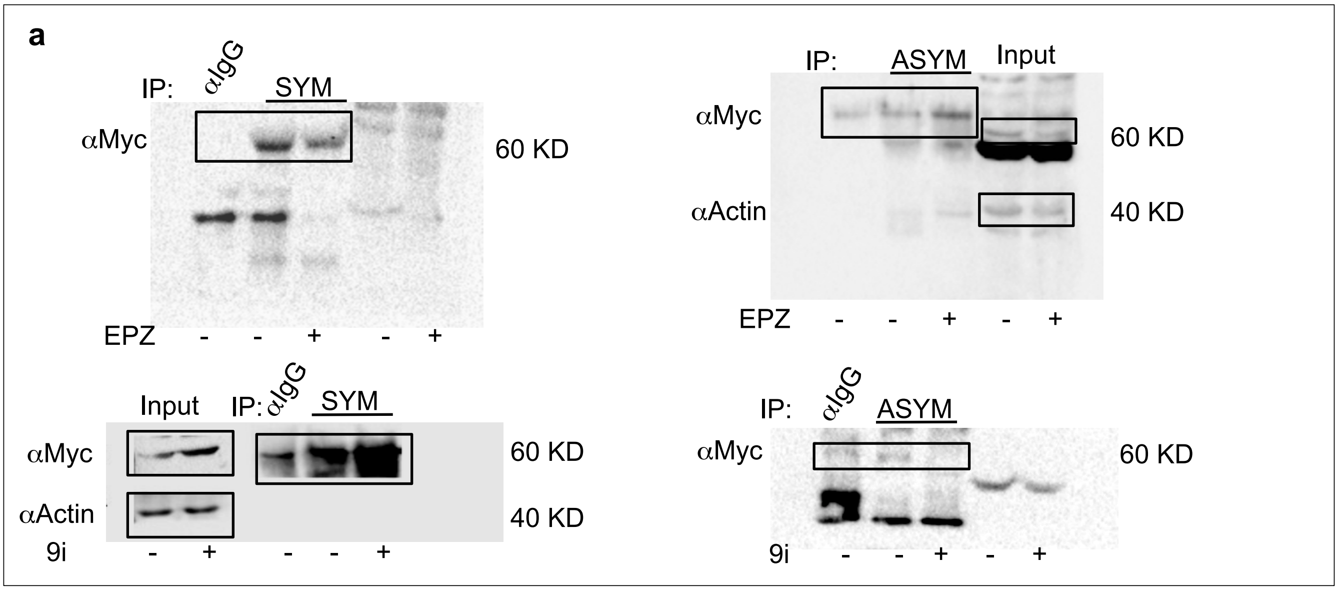

a
IP:
αIgG
SYM
αMyc
60 KD
EPZ - - + - +
IP:
ASYM
Input
αMyc
60 KD
αActin
40 KD
EPZ - - + - +
Input
IP:
αIgG
SYM
αMyc
60 KD
αActin
40 KD
9i - + - - +
IP:
αIgG
ASYM
αMyc
60 KD
9i - - + - +

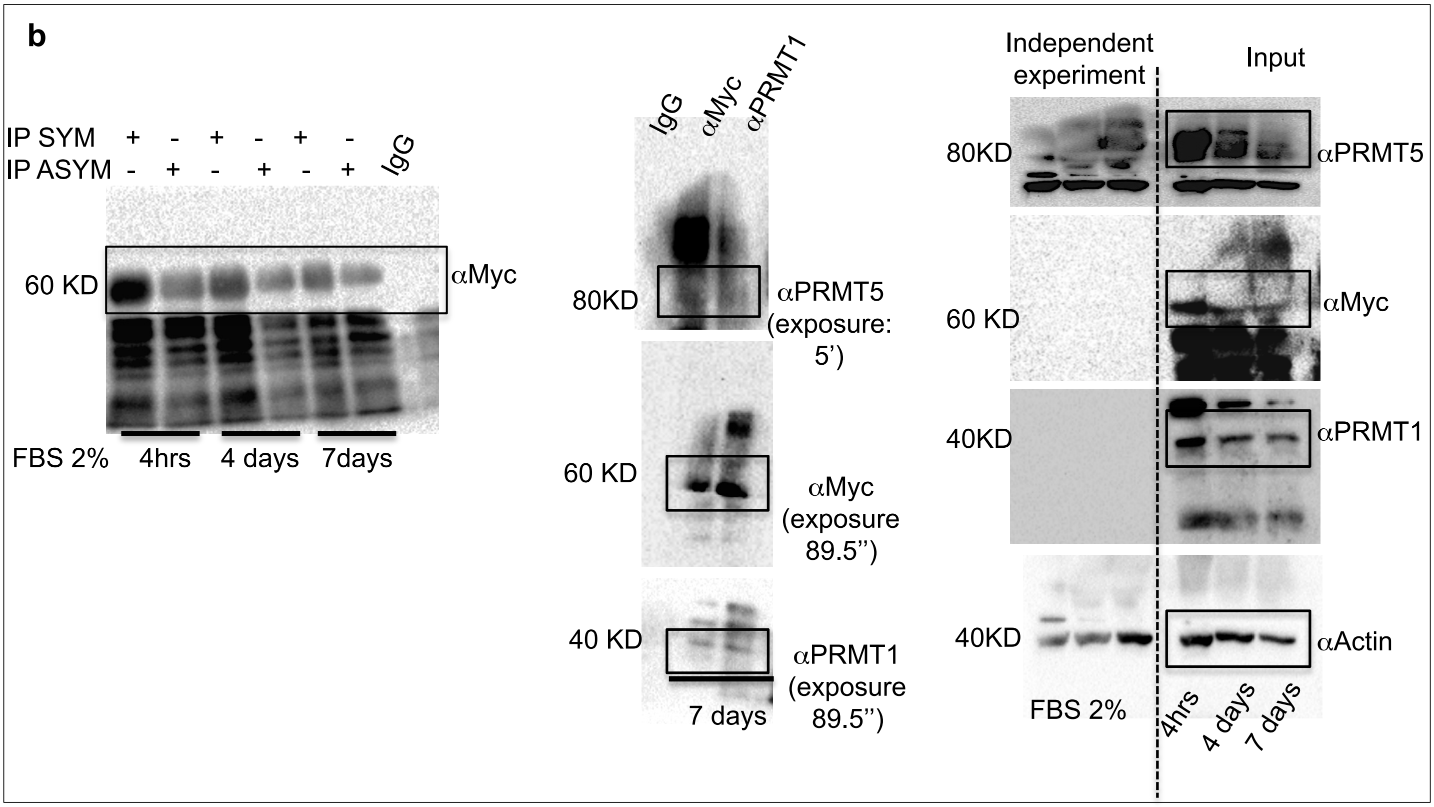

b
IP SYM + - + - + -
IP ASYM - + - + - +
IgG
60 KD
αMyc
FBS 2%
4hrs
4 days
7days
IgG
αMyc
αPRMT1
80KD
αPRMT5 (exposure: 5')
60 KD
αMyc (exposure 89.5'')
40 KD
αPRMT1 (exposure 89.5'')
7 days
Independent experiment
Input
80KD
αPRMT5
60 KD
αMyc
40KD
αPRMT1
40KD
αActin
FBS 2%
4hrs
4 days
7 days

Figure S5

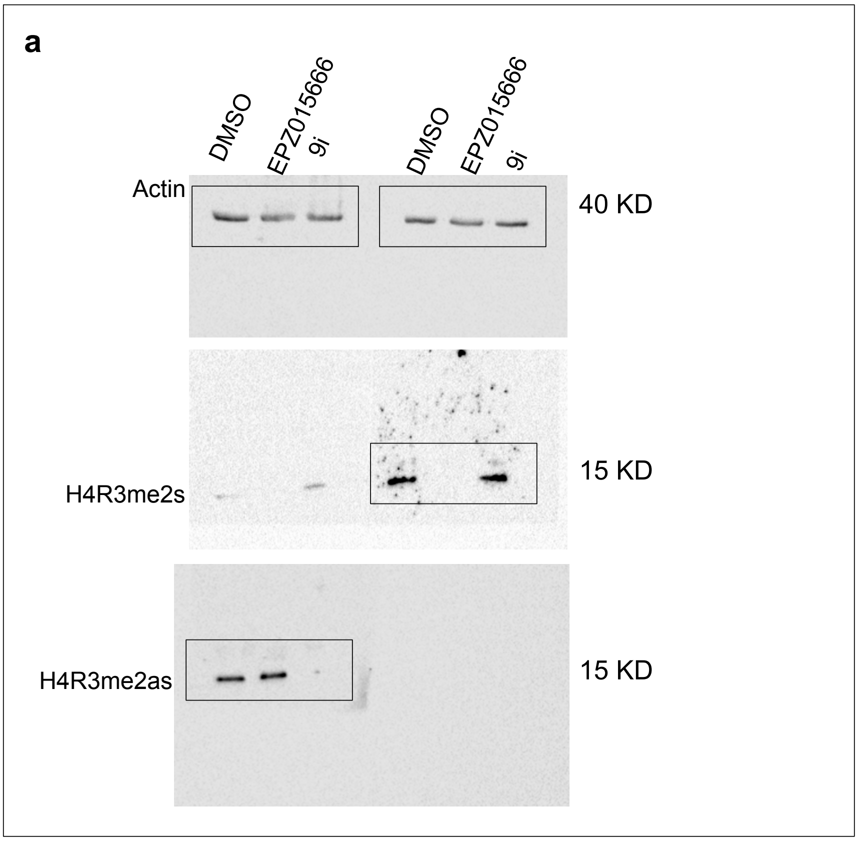
a
DMSO
EPZ015666
9i
DMSO
EPZ015666
9i
Actin
40 KD
H4R3me2s
15 KD
H4R3me2as
15 KD

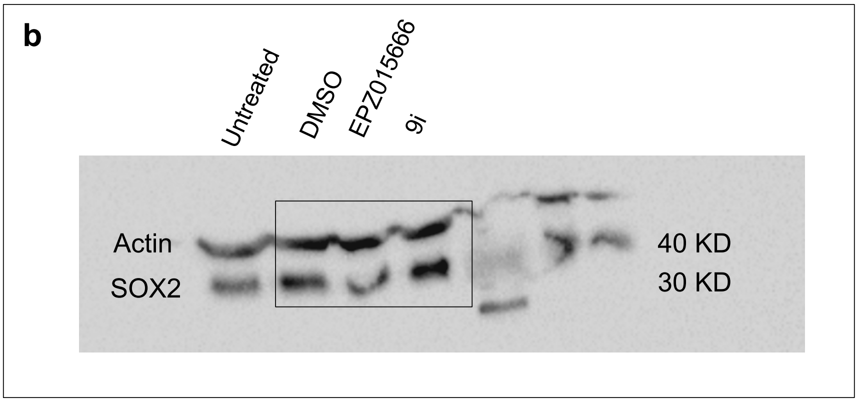
b
Untreated
DMSO
EPZ015666
9i
Actin
SOX2
40 KD
30 KD

Figure S6

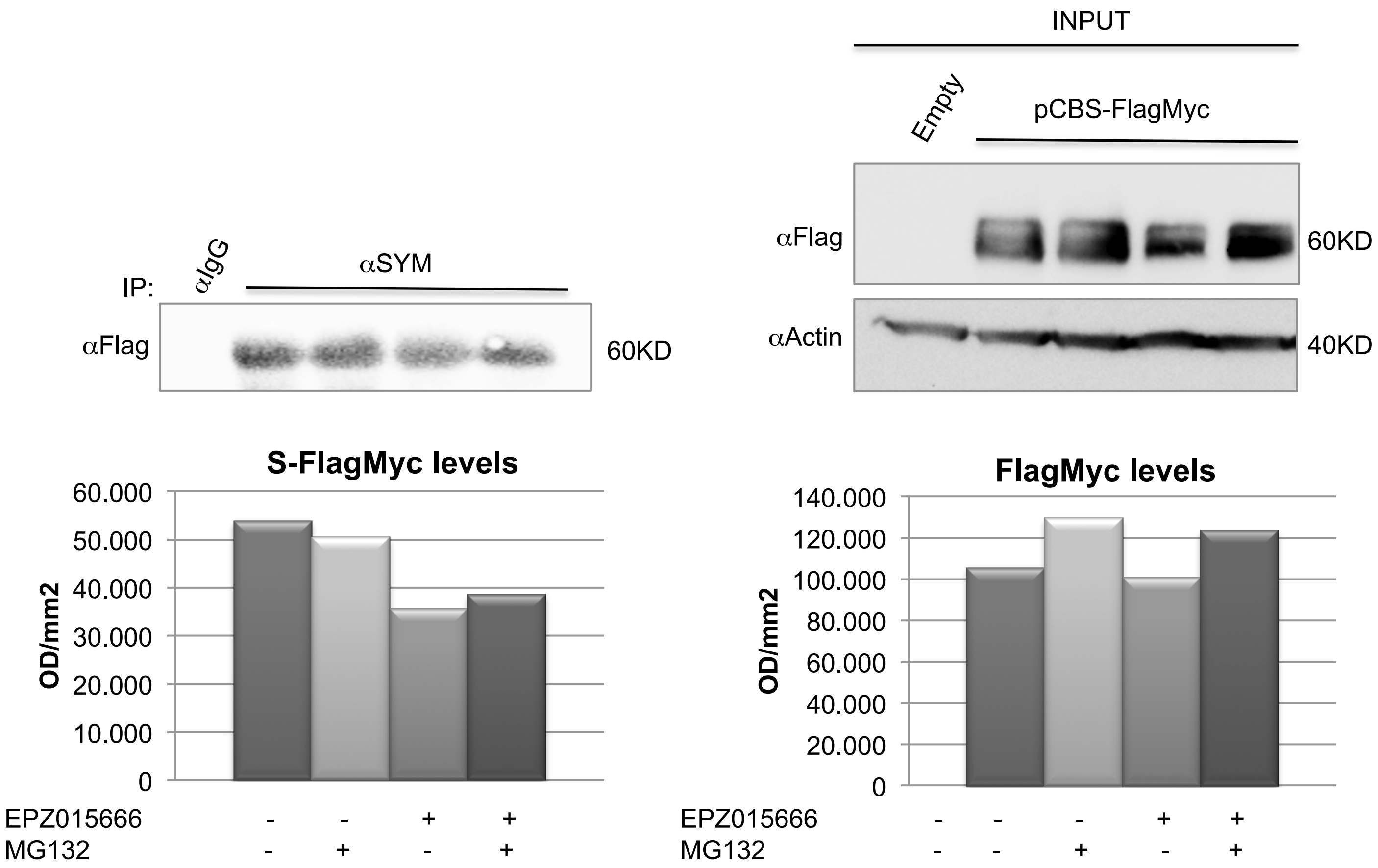
IP:
αIgG
αSYM
αFlag
60KD
INPUT
Empty
pCBS-FlagMyc
αFlag
60KD
αActin
40KD
S-FlagMyc levels
OD/mm2
60.000
50.000
40.000
30.000
20.000
10.000
0
EPZ015666 - - + +
MG132 - + - +
FlagMyc levels
OD/mm2
140.000
120.000
100.000
80.000
60.000
40.000
20.000
0
EPZ015666 - - - + +
MG132 - - + - +

Figure S7

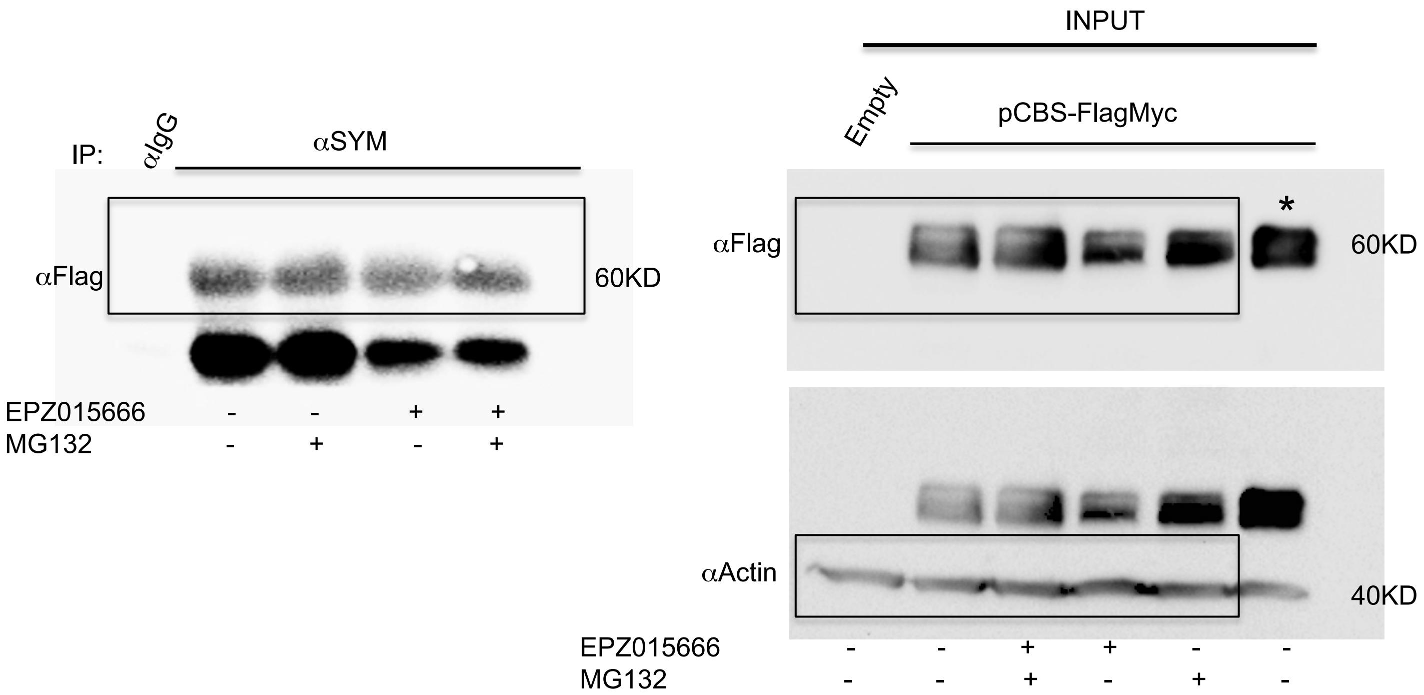

Protein extract from a transfection performed on 26/05/2018
loaded as positive control

MS SREP-19-11671B "The Protein Arginine Methylatransferases 1 and 5 affect Myc properties in glioblastoma stem cells"

Supplementary Figure Legends

Annarita Favia, Luisa Salvatori, Simona Nanni, Lisa K. Iwamoto-Stohl, Sergio Valente, Antonello Mai, Fiorella Scagnoli, Rosaria Anna Fontanella, Pierangela Totta, Sergio Nasi, Barbara Illi.

Figure S6. S-Myc dimethylation decreases upon proteasome inhibition and EPZ015666 treatment.

HEK293T/FlagMyc cells were treated for 24 hours with 5 μM EPZ015666 and the day after with 10 μM MG132 for 6 hours, Thereafter immunoprecipitation experiments were performed with the SYM10 antibody (left). Input is shown on the right. Uncropped images of the blots are shown in supplementary figure S7.

Abbreviations: SYM = SYM10 antibody
